# Characterization of Two Novel Endolysins from Bacteriophage PEF1 and Evaluation of Their Combined Effects on the Control of *Enterococcus faecalis* Planktonic and Biofilm Cells

**DOI:** 10.3390/antibiotics13090884

**Published:** 2024-09-13

**Authors:** Chen Wang, Junxin Zhao, Yunzhi Lin, Su Zar Chi Lwin, Mohamed El-Telbany, Yoshimitsu Masuda, Ken-ichi Honjoh, Takahisa Miyamoto

**Affiliations:** 1 Department of Bioscience and Biotechnology, Graduate School of Bioresource and Bioenvironmental Sciences, Kyushu University, 744 Motooka, Nishi-ku, Fukuoka 819-0395, Japan; wangchen@agr.kyushu-u.ac.jp (C.W.); junxinzhao@hotmail.com (J.Z.); linyunzhi@agr.kyushu-u.ac.jp (Y.L.); suzarchilwin@agr.kyushu-u.ac.jp (S.Z.C.L.); mohamedeltelbany@agr.kyushu-u.ac.jp (M.E.-T.); 2 State Key Laboratory of Food Science and Resources, Nanchang University, Nanchang 330047, China; 3 Department of Bioscience and Biotechnology, Facultuy of Agriculture, Kyushu University, 744 Motooka, Nishi-ku, Fukuoka 819-0395, Japan; y.masuda@agr.kyushu-u.ac.jp (Y.M.); honjoh@agr.kyushu-u.ac.jp (K.-i.H.)

**Keywords:** endolysin, *Enterococcus faecalis*, multidrug-resistance, biofilm, combined effect

## Abstract

Endolysin, a bacteriophage-derived lytic enzyme, has emerged as a promising alternative antimicrobial agent against rising multidrug-resistant bacterial infections. Two novel endolysins LysPEF1-1 and LysPEF1-2 derived from *Enterococcus* phage PEF1 were cloned and overexpressed in *Escherichia coli* to test their antimicrobial efficacy against multidrug-resistant *E. faecalis* strains and their biofilms. LysPEF1-1 comprises an enzymatically active domain and a cell-wall-binding domain originating from the NLPC-P60 and SH3 superfamilies, while LysPEF1-2 contains a putative peptidoglycan recognition domain that belongs to the PGRP superfamily. LysPEF1-1 was active against 89.86% (62/69) of *Enterococcus* spp. tested, displaying a wider antibacterial spectrum than phage PEF1. Moreover, two endolysins demonstrated lytic activity against additional gram-positive and gram-negative species pretreated with chloroform. LysPEF1-1 showed higher activity against multidrug-resistant *E. faecalis* strain E5 than LysPEF1-2. The combination of two endolysins effectively reduced planktonic cells of E5 in broth and was more efficient at inhibiting biofilm formation and removing biofilm cells of *E. faecalis* JCM 7783^T^ than used individually. Especially at 4 °C, they reduced viable biofilm cells by 4.5 log after 2 h of treatment on glass slide surfaces. The results suggest that two novel endolysins could be alternative antimicrobial agents for controlling *E. faecalis* infections.

## 1. Introduction

*Enterococcus faecalis* is a gram-positive bacterium that ubiquitously exists in natural environments, such as water, soil, plants, and sewage, and inhabits the oral cavity and vaginal and intestinal tracts as part of the normal microflora of healthy humans and animals [1,2]. Despite its commensal nature, it was considered an important opportunistic pathogen that causes community- and hospital-acquired infections, including liver damage, urinary tract disorders, bacteremia, and endocarditis [3,4,5]. *E. faecalis* harbors various intrinsic and acquired antibiotic resistance mechanisms, making the treatment of infections extremely difficult, and some multidrug-resistant (MDR) enterococci are becoming increasingly challenging [6,7,8]. Esmail et al. [9] reported a 100% detection rate for MDR *E. faecalis* in hospital-acquired surgical wound infections and bacteremia in Egypt. Gotkowska-Plachta [10] revealed that many enterococcal strains isolated from municipal wastewater are more drug-resistant than those isolated from upstream rivers and hospital wastewater, posing a serious epidemiological threat and risk to public health.

Coupled with the prevalence of MDR strains, strong biofilm formation is a major hazard of *E. faecalis* and has attracted widespread attention. Biofilms are sessile bacterial colonies with an extensive extracellular matrix, found on human tissues, mechanical devices, and other materials [11]. *E. faecalis* is notorious for its strong biofilm-forming ability, as biofilms increase the likelihood of survival in extreme environments [12]. Bacteria in the biofilm have higher antibiotic tolerance than those in their planktonic (liquid culture) counterparts [13]. This phenomenon not only renders the complete eradication of *E. faecalis* biofilms difficult, but also significantly promotes the dissemination of other pathogens, thereby making biofilms a reservoir of antibiotic-resistant genes [14,15]. Several infections associated with *E. faecalis* biofilms have been reported, including in the urinary tract, surgical wounds, intra-abdominal and pelvic, and endocarditis [14]. Thus, biofilm plays an important role in the control of *E. faecalis* infection.

Endolysins, also known as phage lysins, are enzymes encoded by bacteriophages that hydrolyze the peptidoglycan of the bacterial cell wall during the final stages of the phage replication cycle, enabling the release of progeny phage particles [15]. Compared with phages, endolysins have many advantages as potential antimicrobial agents. Several phage endolysins exhibit a broader lytic spectrum than their host phages and can directly target specific peptidoglycan bonds on the cell surface, leading to rapid bacterial cell lysis within minutes of exposure [11,16]. As endolysins are independent of active host metabolism, antimicrobial resistance mechanisms hardly evolve [17]. Moreover, unlike phages that have limited applications due to the presence of toxic residues in their lysates, endolysins have better safety profiles as antibacterial agents because they are naturally occurring proteins [18].

Over the past decade, the biocontrol potential of several endolysins has been documented against *E. faecalis*. Zhang et al. [19] reported that LysIME-EF1, which contains an N-terminal CHAP and a C-terminal cell wall-binding domain (CBD), had a wider bactericidal spectrum than the phage against its host, including two vancomycin-resistant *E. faecalis* strains. A similar structure was found in LysPEF-P10, which reduced the abundance of the genus *Enterococcus* in the feces of normal mice with high efficiency [20]. Zhang et al. [21] reported that endolysin ORF28 acted as an N-acetylmuramidase, endo-β-N-acetylglucosaminidase, and endopeptidase, rather than an N-acetylmuramoyl-L-alanine amidase as originally predicted. However, the types of endolysins discovered in *E. faecalis* are limited, and little work has been done to evaluate the antimicrobial ability of multidrug-resistant strains and specifically determine their antibiofilm activity. In this study, two novel endolysins encoded by the genomic DNA of *E. faecalis* phages were characterized, and their antibacterial activity was evaluated against both planktonic and biofilm cells of MDR and strong biofilm-forming *E. faecalis* strains.

## 2. Results

### 2.1. Identification and Overexpression of Recombinant Endolysin

**Figure 1:**
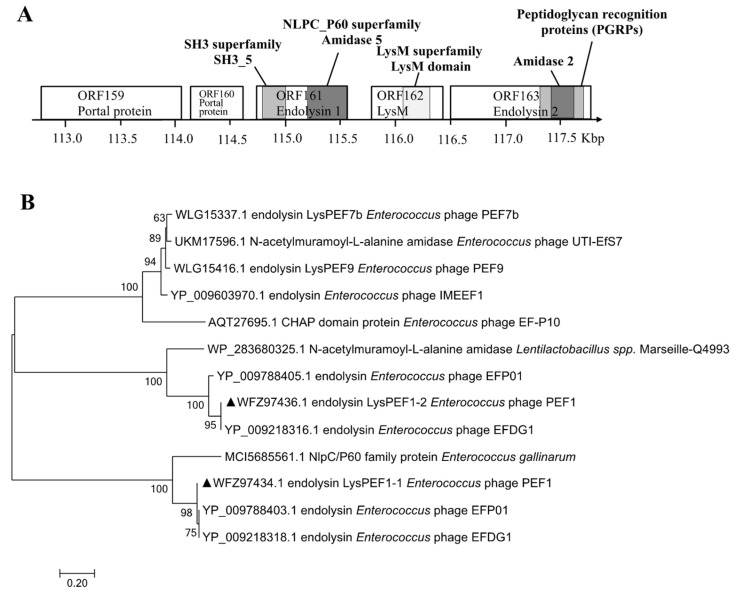

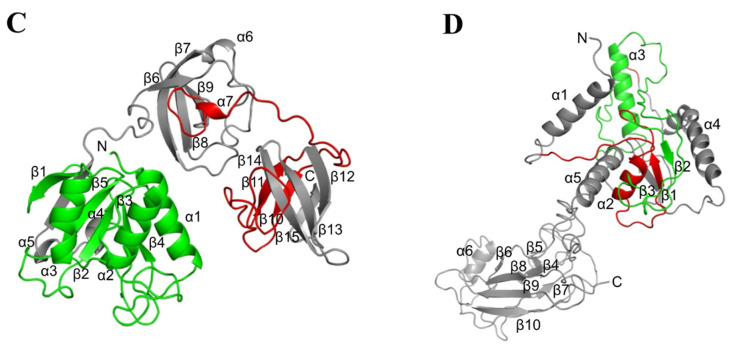
Endolysins LysPEF1-1 and LysPEF1-2 derived from bacteriophage PEF1. (**A**) Schematic representation of phage PEF1 lysis gene module (ORFs 159 to 163). Light and dark gray in ORF161 and ORF163 columns represent the localization of the endolysin cell binding domain and catalytic domains, respectively. Pale gray represents the localization of the transmembrane helices in ORF162 LysM structure. (**B**) Phylogeny of endolysins LysPEF1-1 and LysPEF1-2 by using Neighbor-Joining method (marked with “▲” symbols). Scale bar indicates the percentage of statistical support. Ultrafast bootstrap support percentages are indicated adjacent to the nodes. Tip labels include NCBI accession numbers and corresponding phage names for the respective endolysin proteins. Three-dimensional structure of the endolysin LysPEF1-1 (**C**) and LysPEF1-2 (**D**) was prepared by PyMOL. Green color region: enzymatic active domains; Red color region: cell well-binding domains; Gray color region: hypothetical protein domains. α-helices and β-strands were marked sequentially.

As shown in Figure S1, the phage PEF1 genome was approximately 150 kb long, encoding 256 open reading frames (ORFs). According to the gene module alignment results, ORF161–ORF163 are located within the gene module of the PEF1 genome, the function of which was predicted to be related to cell lysis (Figure 1A). ORF161 consisted of 873 nucleotides, suggesting that the gene product is an endolysin protein (LysPEF1-1) with 290 amino acids and a molecular mass of 31.5 kDa. LysPEF1-1 was predicted to contain an enzymatically active domain (EAD) in the amino acid sequence position 3–129, belonging to the NLPC_60 superfamily (pfam05382; E-value, 3.75e−34), and a cell-binding domain (CBD) in the amino acid sequence position of 220–278, belonging to the SH3 superfamily (pfam08460; E-value, 1.93e−09). The deduced ORF163 (1257 bp) encoded another putative endolysin protein (LysPEF1-2) consisting of 448 amino acids with a molecular weight of 45.8 kDa. LysPEF1-2 endolysin contained a conserved peptidoglycan recognition protein (PGRP) domain at amino acid sequence position 28–157, which belonged to the PGRP superfamily (cd06583; E-value, 1.57e−19). Several highly conserved residues were identified within this domain, such as H145 and C153, which were likely involved in forming a Zn^2+^ binding site. A58, H145, T151, and C153 were recognized as amidase catalytic sites. Additionally, residues A54, A58, V71, F78, H79, N95, H145, T149, T151, A152, and C153 were predicted to contribute to substrate recognition and binding.

The secondary structure and disorder regions of two endolysins were predicted and shown in Figure S2, with α-helices, β-strands, and disordered regions color-coded by confidence level. Three-dimensional structure alignment results for LysPEF1-1 and LysPEF1-2 were shown in Table S2 and Table S3, respectively. A confidence summary of the predicted model by residue indicates that 216 residues (74%) of LysPEF1-1 were modeled with 99.6% confidence using the highest scoring template, while 145 residues (35%) of LysPEF1-2 were modeled with 100% confidence. The predicted three-dimensional structures of LysPEF1-1 and LysPEF1-2 are shown in Figure 1C,D. The regions in green, red, and grey represent the predicted enzymatic active domains, cell wall-binding domains, and hypothetical protein domains of the two endolysins, respectively. 

Although LysPEF1-1 and LysPEF1-2 were identified as amidase-type enzymes that hydrolyze the peptidoglycan bond between muramic acid and the peptide, phylogenetic analysis showed that they appeared in different branches with a high bootstrap value (100), indicating a low degree of homology (Figure 1B).

The two endolysin genes were amplified using PCR and ligated into the pET28a (+) vector, and the recombinant proteins LysPEF1-1 and LysPEF1-2 with 6 × His-Tag at their N-terminal were overexpressed in *E. coli* BL21 (DE3). After purification via affinity chromatography, a thick single band was obtained on the SDS-PAGE gel, with a size between 25–35 kDa and 40–45 kDa for LysPEF1-1 and LysPEF1-2, respectively, which was consistent with the predicted sizes (Figure S3A,B).

### 2.2. Lytic Activities of Recombinant LysPEF1-1 and LysPEF1-2

**Figure 2:**
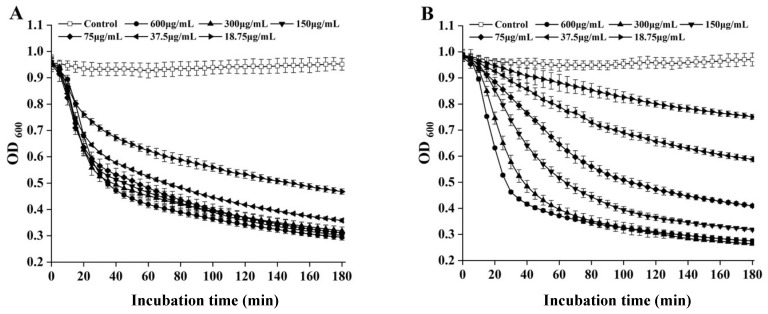
The lytic activity of endolysins LysPEF1-1 (**A**) and LysPEF1-2 (**B**). The lytic activity of recombinant endolysins LysPEF1-1 and LysPEF1-2 at different concentrations against *Enterococcus faecalis* JCM 7783^T^ at 25 °C. The error bars indicate the standard error of the mean (n = 3).

Turbidity reduction assays were performed to determine the lytic activity of LysPEF1-1 and LysPEF1-2. Both recombinant LysPEF1-1 and LysPEF1-2 showed strong lytic activity against *E. faecalis* JCM 7783^T^, with the largest OD_600_ reduction in 0.72 (73%) within 3 h for 300 μg/mL LysPEF1-2 (Figure 2B). LysPEF1-1 showed similar reduction curves at the concentration range of 75–600 μg/mL. However, in LysPEF1-2, the lytic activity increased in a dose-dependent manner.

**Figure 3:**
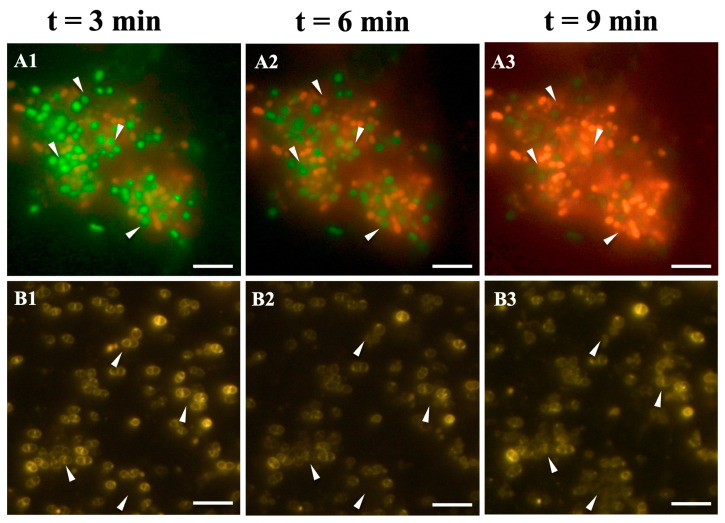
Visualization of the lytic activity of endolysin LysPEF1-1 on *E. faecalis* JCM 7783^T^. Exponentially growing cells were stained by LIVE/DEAD™ Sperm Viability Kit (**A1**–**A3**) and bacterial membrane-detecting probe POLARIC-500BCS (**B1**–**B3**) subsequently mixed with 150 μg/mL LysPEF1-1. The mixture was dropped onto a poly-L-lysine glass slide and covered with a coverslip and monitored. Three-minute intervals are shown for t = 3, 6, and 9 min (the first measurement started at 3 min after adding endolysin). The live cell is shown as a green color and the dead cell is shown as a red color. White arrowheads in the photos indicate cells at the same location at different treatment times for each stain. Scale bar = 10 µm.

Morphological changes of *E. faecalis* cells in the presence of 150 μg/mL LysPEF1-1 were observed using fluorescence microscopy (Figure 3). As indicated by the white arrowhead in Figure 3A1–A3, the color of the cells changed from green to orange with increasing treatment time, indicating decreased viability and membrane damage. In contrast, all cells in the control group survived (Figure S5). Moreover, as shown in Figure 3B1–B3, the clear cytoplasmic membrane was blurred with increasing treatment time, suggesting endolysin-induced membrane damage.

### 2.3. Characterization of Recombinant Endolysins LysPEF1-1 and LysPEF1-2

**Figure 4:**
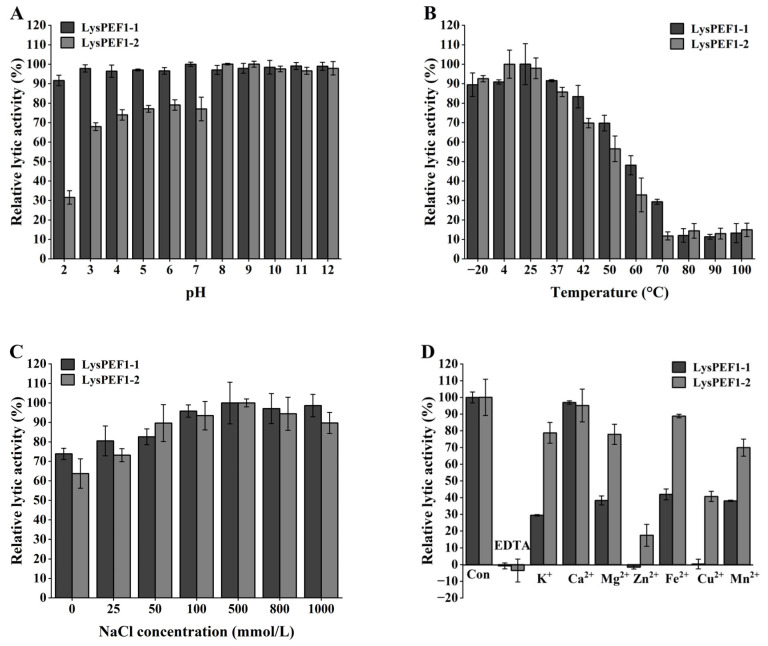
Effects of various environmental factors on the lytic activity of recombinant LysPEF1-1 and LysPEF1-2. Lytic activity of LysPEF1-1 and LysPEF1-2 (150 μg/mL) against *E. faecalis* JCM 7783^T^ was determined after the treatments of (**A**) pH, (**B**) temperature, (**C**) NaCl concentration, and (**D**) presence of metal ions. The error bars show the standard error of the mean (n = 3).

As shown in Figure 4A, LysPEF1-1 exhibited high lytic activity after being treated with a wide pH range of 3–12, only with a 10% decrease at pH 2. In contrast, LysPEF1-2 maintained high lytic activity only after treatment at alkaline pH values (8–12). However, with a decrease of approximately 30% at pH from 3 to 7 and a 70% reduction at pH 2, compared to its activity at pH 8. 

The thermal stabilities of the two endolysins were investigated after incubation at different temperatures for 1 h (Figure 4B). Both of the endolysins retained their lytic activity above 90% at temperatures ranging from −20 to 37 °C. The highest stability of LysPEF1-1 and LysPEF1-2 was observed at 25 and 4 °C, respectively. However, the stability decreased with increasing temperature. Approximately 10% lytic activity was retained for both endolysins when incubated at 80 °C.

Both endolysins exhibited strong lytic activity after being treated with a wide range of NaCl concentrations (1–1000 mmol/L), with the highest stability observed at 500 mmol/L NaCl. The lytic activity decreased only by 25 and 35% for LysPEF1-1 and LysPEF1-2 at 0 mmol/L treatment, respectively (Figure 4C). 

The effects of metal ions on the lytic activity of the two endolysins are shown in Figure 4D. The large decrease in the lytic activity of both endolysins after being treated with 4 mmol/L EDTA suggests that metal ions are essential for the activity of both endolysins. The lytic activities of both endolysins were significantly recovered (*p* < 0.05) by the addition of K^+^, Ca^2+^, Mg^2+^, Fe^2+^, or Mn^2+^. However, addition of Zn^2+^ and Cu^2+^ did not increase the lytic activity of LysPEF1-1 decreased by EDTA. Among these metal ions, Ca^2+^ was the most effective for the recovery of lytic activity of both endolysins, recovering up to 90% of the initial activity.

### 2.4. Lytic Spectra of Recombinant LysPEF1-1 and LysPEF1-2

As shown in Table 1, LysPEF1-1 showed a wider lytic spectrum against 89.86% (62/69) of *Enterococcus* spp. tested than that of the phage PEF1, 84.06% (58/69). At a final concentration of 40 µg/mL, LysPEF1-1 exhibited strong lytic activity (lytic activity > 50%) against 62.32% (43/69) of the *Enterococcus* strains, significantly surpassing LysPEF1-2, which showed strong activity against only 4.35% (3/69) of the strains. The highest lytic activities of LysPEF1-1 and LysPEF1-2 were expressed on *E. faecalis* E26 and J28; their initial OD_600_ reduced by 81.23% and 70.66%, respectively. 

In addition, both endolysins exhibited lytic activity against two gram-positive bacteria, *Listeria monocytogenes* and *Clostridium perfrigens*, and eight other gram-negative bacteria pretreated with chloroform. Notably, both endolysins demonstrated strong lytic activity against *Campylobacter jejuni* and *C. coli* strains.

### 2.5. Effects of Recombinant Endolysins on the Viability of E. faecalis Planktonic Cells

**Figure 5:**
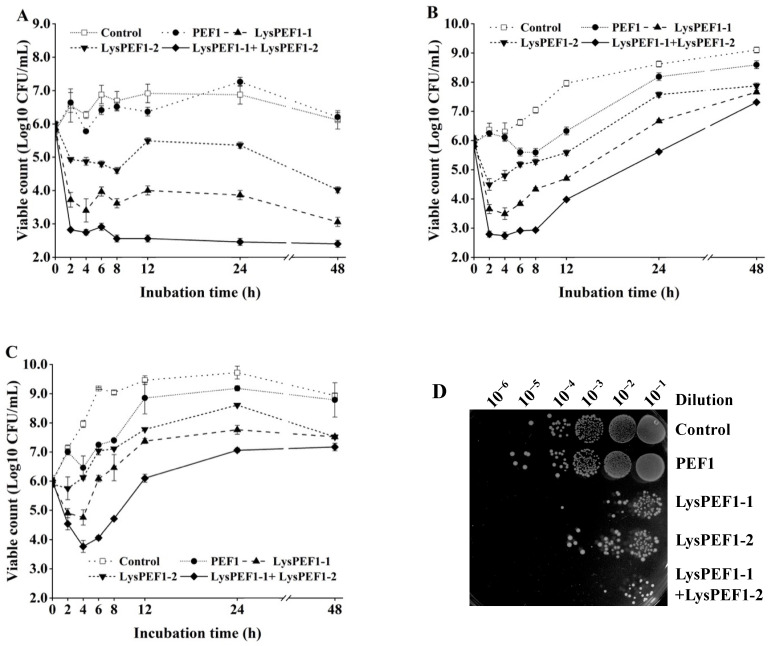
Effects of endolysins LysPEF1-1 and LysPEF1-2 on the viability of *E. faecalis* wild-type strain E5 in broth. *E. faecalis* E5 was incubated alone or with single phage PEF1, LysPEF1-1, LysPEF1-2, and equal mixture of LysPEF1-1 and LysPEF1-2 at (**A**) 4 °C, (**B**) 25 °C, (**C**) 37 °C. The error bars indicate the standard error of the mean (n = 3). (**D**) Survival test of *E. faecalis* E5 on TSA agar dishes after the cells were lysed by phage PEF1, LysPEF1-1, LysPEF1-2, or mixture of LysPEF1-1 and LysPEF1-2 at 37 °C for 2 h.

As shown in Figure 5, the endolysins LysPEF1-1 and LysPEF1-2 were tested alone and in combination on the viability of *E. faecalis* multidrug-resistant strain E5 in broth. In the absence of endolysins or phage, the viable counts of the strain E5 increased from 6 log CFU/mL to approximately 6.5, 9, and 8.5 log CFU/mL at 4, 25, and 37 °C, respectively, after 48 h incubation. In the presence of a single endolysin, the viable count of *E. faecalis* E5 sharply decreased within 2 h. The largest decrease was detected by the treatment with LysPEF1-1 (by 2.2 log, 2.3 log, and 1.1 log at 4, 25, and 37 °C, respectively). However, from 4–48 h of incubation, the viable count of E5 planktonic cells in the single endolysin-treated groups increased to varying degrees.

Compared with single endolysin, the combination of two endolysins showed stronger lytic activity than those of the single use. The viable counts of E5 decreased after 4 h incubation by 3.1 log, 3.2 log, and 2.2 log at 4, 25, and 37 °C, respectively. Especially at 4 °C (Figure 5A), the combination of the two endolysins reduced the viable count by 3.5 log after 8 h of incubation, and no regrowth was observed until 48 h.

### 2.6. Effects of Recombinant Endolysins on Biofilm Formation of E. faecalis

**Figure 6:**
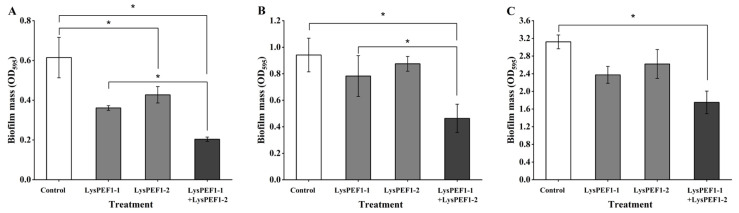
Effects of endolysins on biofilm formation of *E. faecalis* JCM 7783^T^. *E. faecalis* JCM 7783^T^ was incubated with LysPEF1-1 and/or LysPEF1-2 at a final concentration of 150 μg/mL for 48 h at 4 °C (**A**), 25 °C (**B**), and 37 °C (**C**). The error bars indicate the standard error of the mean (n = 3); *, *p* < 0.05.

The effects of the two endolysins, alone and in combination, on biofilm formation by *E. faecalis* JCM 7783^T^ were assessed in 96-well microplates (Figure 6). After 48 h incubation, the biofilm mass was determined by the crystal violet staining method and presented as absorbance at 595 nm (OD_595_). The OD_595_ values of the control wells without endolysins were 0.62, 0.94, and 3.1 at 4, 25, and 37 °C, respectively. The single use of LysPEF1-1 demonstrated stronger inhibitory activity against biofilm formation compared to LysPEF1-2. However, when the two endolysins were combined, the inhibition in biofilm formation mass was significantly greater than those by the single use, with decreases by 0.16, 0.32, and 0.62 at 4, 25, and 37 °C, respectively, compared to those of the LysPEF1-1 alone. This indicates that the combination of the two endolysins was the most effective.

### 2.7. Effects of Recombinant Endolysins on Viability of E. faecalis in Biofilm on Different Surfaces

**Figure 7:**
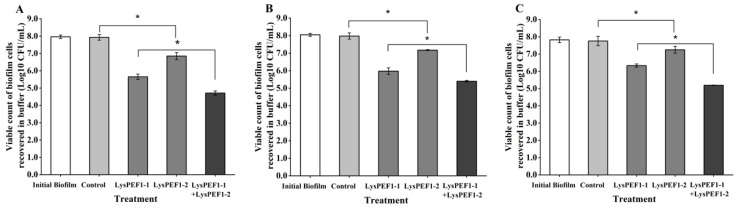

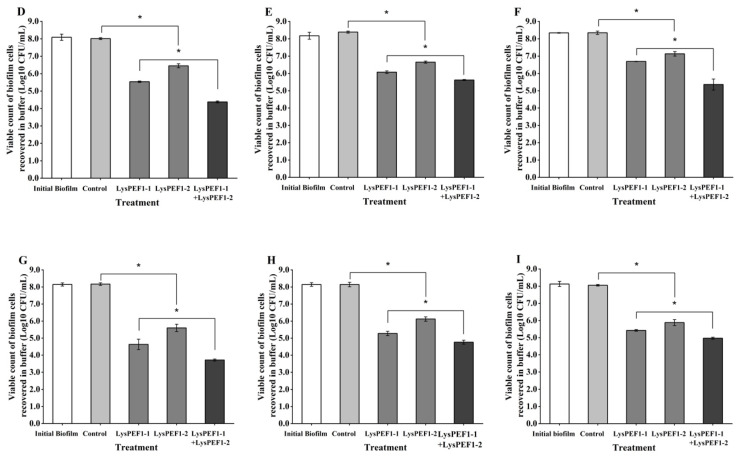
Effects of LysPEF1-1 and/or LysPEF1-2 on reduction of biofilm on different surface materials. Mature *E. faecalis* JCM 7783^T^ biofilm cells were incubated with LysPEF1-1, LysPEF1-2, and mixture of LysPEF1-1 and LysPEF1-2 (10^9^ PFU/mL) on 96-well polystyrene plates at 4 °C (**A**), 25 °C (**B**), and 37 °C (**C**); on 304 stainless steel surfaces at 4 °C (**D**), 25 °C (**E**), and 37 °C (**F**); on glass slide surfaces at 4 °C (**G**), 25 °C (**H**), and 37 °C (**I**). The error bars indicate the standard error of the mean (n = 3); *, *p* < 0.05.

The efficacy of two endolysins alone or in combination in reducing the viability of biofilm cells of JCM 7783^T^ was tested on 96-well polystyrene plates (Figure 7A–C), 304 stainless steel surfaces (Figure 7D–F), and glass slide surfaces (Figure 7G–I) at 4, 25, and 37 °C. The initial viable counts recovered from the biofilms on each surface were approximately 8.0 log10 CFU/mL. After 2 h of treatment with endolysins, especially in combination, the viable counts of biofilm cells were significantly reduced (*p* < 0.05). In the biofilm on 96-well plates, viable counts of biofilm cells were reduced by 3.2, 2.5, and 2.6 log, respectively, after the combined treatment at 4, 25, and 37 °C for 2 h. The same trend was observed in the experiments on the 304 SSC and glass slide surfaces. In addition, compared to the biofilms on the surfaces of 96-well plates and 304 SSCs, endolysins had the strongest effect on the biofilm cells of *E. faecalis* on the glass slide surface. Especially at 4 °C, the combination of LysPEF1-1 and LysPEF1-2 reduced the viable cells by 4.5 log (Figure 7G). The reduction in viability was 1.9 and 0.9 log lower, respectively, than those of the single treatments with LyPEF1-1 and LysPEF1-2, suggesting an additional effect of the two endolysins on the reduction of viability of *E. faecalis* in biofilm.

## 3. Discussion

The application of bacteriophage-encoded endolysins is considered one of the best alternatives to antibiotics against pathogenic *E. faecalis* infections, particularly those caused by multidrug-resistant strains [15,22]. In this study, two new endolysins, LysPEF1-1 and LysPEF1-2, derived from the lytic phage PEF1, were successfully overexpressed in *E. coli* and characterized in detail. Nucleotide sequences and phylogenetic analyses revealed that the two endolysins are homologous to two N-acetylmuramoyl-L-alanine amidase genes encoded in *Enterococcus* phages EFDG1 (Accession NC_029009.1) and EFP01 (Accession: NC_047796.1). However, the proteins encoded by these endolysin genes have not been expressed or characterized, and their antibacterial effects on their hosts have not been evaluated. Among the previous reports, the optimal pH environments of two endolysins from *E. faecalis*, PlyV12 [23] and LysEF-P10 [24], were reported to be approximately 6.5 and 7, respectively, and their activity was greatly affected by pH. In this study, LysPEF1-2 showed high activity (above 90%) under alkaline pH conditions (8–12), while LysPEF1-1 maintained high activity in both acidic and alkaline pH ranges (2–12), demonstrating its strong pH tolerance, though the molecular mechanism underlying it needed to be further understood. According to Figure 4B, 4 and 25 °C were the optimal temperatures for LysPEF1-1 and LysPEF1-2, and their lytic activities were significantly affected by the treatment at above 50 °C (relative activity lower than 50%). Similar favorable protein properties at lower temperatures have been reported for the endolysins CHAPk-SH3bk from *Staphylococcus aureus* [25] and LysCPAS15 from *Clostridium aerogenes* [26]. After inhibiting the activity of the two endolysins with EDTA, the addition of Ca^2+^ was most effective in restoring the cleavage activity of the endolysins at 1 mmol/L, which may be explained by the increased interaction of the endolysins with the peptidoglycan backbone mediated by Ca^2+^ metal ions [27]. However, 1 mmol/L Zn^2+^ and Cu^2+^ did not effectively restore the activity of LysPEF1-1. The reason might be that Zn^2+^ and Cu^2+^ are not essential for the lytic activity of the NlpC/P60 family’s amidase. Unlike the Zn^2+^-binding sites that have been demonstrated in the EAD domain of the PGRP family present in LysPEF1-2, the amidase of the NlpC/P60 family typically relies on conserved catalytic triad residues, such as Cysteine, Histidine, and Glutamate/Aspartate, to exert its lytic activity. These enzymes generally achieve catalysis through interactions between these residues and the substrate, rather than depending on metal ions [28]. Similar findings were observed for the *E. faecalis* endolysin, LysIME-EF1 [29]. Subsequent mutagenesis experiments further confirmed that these residues are necessary for catalytic activity [25]. 

LysPEF1-1 had a broader host range than PEF1, as shown by the lysis of three additional strains of tested *Enterococcus faecium* and two gram-positive bacteria, *L. monocytogenes* No. 185 and *C. perfrigens* JCM 1290^T^ and S1 (Table 2). A similar phenomenon was observed in *Streptococcus agalactiae* endolysin EN534-C [30] and *Streptomyces avermitilis* LytSD [31], both of which showed lytic activity against a variety of gram-positive strains. This may be explained by the fact that although gram-positive bacteria may have variations in cell wall components, there are shared features, such as teichoic acids, lipoteichoic acids, and other surface-exposed molecules [32]. The conserved domains of endolysins, which are shared across endolysins derived from various genera, allow them to recognize these common elements and bind to and exert their lytic activity against different gram-positive species [11,15]. Moreover, it is noteworthy that both endolysins were effective in lysing *C. jejuni* L26, *C. coli* Can 10, *S.* Typhimurium IFO 12529, and *P. alcaligenes* NBRC 14,159 after the cell walls were permeabilized by chloroform treatment, despite their CBDs belonging to different families. This suggests a great potential for combining these endolysins with biocontrol agents that damage the outer membrane of Gram-negative bacteria.

In the determination of enzyme activity, when the concentration of LysPEF1-1 is higher than 35.7 μg/mL, the OD_600_ value is reduced to approximately 30% of the initial value within 3 h. Increasing protein concentration has little effect on the cleavage potential of endolysin. Previously, Ply12 endolysin from *E. faecalis* also reduced the OD_600_ to approximately 25% of the initial value at above 25 μg/mL and did not increase with increasing concentration [24]. Similar endolysin properties were also observed for previously expressed *E. faecalis* endolysin ORF9 [33] and *C. perfringens* endolysin LysCP28 [34]. The modest dose-dependent lytic activity of endolysin may be explained by the saturation of the binding sites on the bacterial cell surface, which limits the accessibility of endolysin to its substrate, peptidoglycan [18,35]. In contrast, although LysPEF1-2 reduced the OD_600_ value to approximately 27% of its initial value at its maximum, its lytic activity was dose dependent (18.75 to 600 μg/mL). The molecular weight of LysEPF1-2 is relatively larger than that of LysPEF1-1, which may hinder its ability to efficiently access and interact with the peptidoglycan layer for cell lysis, potentially due to steric hindrance or reduced diffusion through the thick peptidoglycan structure of Gram-positive cells [36]. This might also explain why the bactericidal effect of LysPEF1-2 at the same concentration (150 μg/mL) was not as good as that of LysPEF1-1 against *E. faecalis* planktonic cells and biofilm cells.

In this study, two endolysins exhibited strong bactericidal ability against the multidrug-resistant strain, *E. faecalis* E5, reducing the viable counts by 1.2 and 2.2 logs at 4 and 25 °C, respectively, within 2 h. In contrast, the phage PEF1, from which the endolysins were derived, did not lyse the strain E5. Phages typically exhibit high specificity for infecting their host bacterial strains during the lytic cycle [37]. However, endolysin does not rely on the phage replication cycle and acts directly on bacterial cell walls [22]. Therefore, this is the reason to explain that endolysins have a wider host range than phages, which significantly reduces the likelihood that host cells will develop resistance to endolysins. Cheng et al. [20] reported that LysEF-P10 exhibits bactericidal activity against multidrug-resistant strains of *E. faecalis*. The combination of endolysin Ply2660 and cathelicidin LL-37 decreases the viability of multidrug- resistant *E. faecalis* V583 by 3.6 log [15]. These results demonstrate the potential of endolysin as an alternative antibacterial agent for the treatment of multidrug-resistant *E. faecalis*; however, further experiments, particularly involving human-derived multidrug-resistant enterococci, are needed to confirm its effectiveness in treating human infections.

In the biofilm formation inhibition assay, individual endolysin treatments at 4 °C resulted in a significant decrease in the biofilm mass compared to the control group, while no significant differences were observed at 25 °C and 37 °C. The amount of biofilm mass in the control was greater at higher incubation temperatures. This is because the bacterial growth rate was faster at 25 and 37 °C, where the metabolic activity of bacteria was higher than that at 4 °C, leading to a greater production of extracellular polymeric substances required for biofilm formation. As shown in Figure 5, at 25 and 37 °C, while some bacterial cells were killed by the endolysins, the survived population continued to grow, and the damaged cells recovered and proliferated, resulting in a viable count recovery at 48 h. This can be explained by the higher viable counts and complex biofilm environment formed at 25 and 37 °C after 48 h of incubation, which may impose restrictive conditions on the lytic activity of the endolysins [38]. In the control of *E. faecalis* JCM 7783^T^ biofilm, the combination of LysPEF1-1 and LysPEF1-2 showed a stronger effect than single endolysin treatments at a comparable dose. Two classes of endolysins, N-acetylmuramyl-L-alanine amidase (AmiA) and β-N-acetylglucosaminidase (GlcA), have been reported to have a synergistic bactericidal effect on staphylococci, because GlcA activity required the prior removal of the stem peptide by AmiA for its activity [39]. In this study, although the EADs (from the NLPC family) of LysPEF1-1 and the PGRP structure of LysPEF1-2 were both predicted to belong to the amidase enzyme family, they still showed certain discrepancies in enzymatic activity and recognition mechanisms [40,41]. This was further supported by the stronger antibacterial activity of chimeric endolysin, which contains two catalytic regions from the NLPC and PGRP families, against *S. aureus*, *S. epidermidis*, and *Enterococcus* [42]. In addition, Liu et al. [43] revealed that the SH3 domain of Ct-PGRP-S1, a short-type peptidoglycan-recognition protein that originates from *Charonia tritonis*, facilitates the interaction of PGRP with peptidoglycans on the cell wall and exerts immune inhibition. This may be explained by the essential role of the Src homology 3 (SH3) domains in the lytic activity of endolysins, acting as the cell wall-binding domain and recognizing peptide side chains to properly orient the catalytic domain for binding to the glycan backbone [44,45,46]. Further studies are required to fully understand the mechanism of cooperation between these two endolysins. The combined use of LysPEF1-1 and LysPEF1-2 appeared to exhibit better performance than the equivalent use of a single endolysin in both inhibiting biofilm formation by *E. faecalis* and removing *E. faecalis* biofilm cells from the surfaces of different materials. This suggests that they may serve as alternative natural antimicrobial agents against *E. faecalis* biofilms in food processing, equipment piping cleaning, and other applications.

## 4. Materials and Methods

### 4.1. Phages, Bacteria, and Growth Conditions 

The bacteriophage PEF1 is an enterococcal siphovirus isolated in a previous study [47]. The bacterial strains used in this study are listed in Table S1. Fifty-nine *E. faecalis* and eight other *Enterococcus* spp. were previously isolated and identified from food sources in Japan and Egypt and stored in Microbank^TM^ (Pro-Lab Diagnostics, Richmond Hill, ON, Canada) at −80 °C. *Enterococcus faecalis* JCM 7783^T^ was purchased from the Japan Collection of Microorganisms (RIKEN, Saitama, Japan). Plasmid pET-28a (+) vectors carrying an N-terminal His-tag (Novagen, Madison, WI, USA) were used for the recombinant protein cloning experiments. *Escherichia coli* BL21 (DE3) cells were purchased from New England Biolabs (Tokyo, Japan) and used as the host for protein expression. *E. faecalis* JCM 7783^T^ and multidrug-resistant *E. faecalis* E5 (resistant to erythromycin, gentamicin, kanamycin, rifampin, vancomycin, and ciprofloxacin) were used as hosts for the characterization of the antimicrobial effects of the recombinant proteins in vitro. Each strain stored in Microbank^TM^ at −80 °C was streaked onto Tryptic Soy Agar (TSA; Becton, Dickinson and Company, Sparks, MD, USA) plates and maintained at 4 °C until use. 

### 4.2. Bioinformatic Analysis

The genomic DNA of phage PEF1 was sequenced, annotated, and submitted to GenBank under the accession number OQ653963 [47]. A National Center for Biotechnology Information (NCBI) Conserved Domain Search was used to predict the domain architecture of endolysins, and the NCBI Protein Basic Local Alignment Search Tool (BLASTP) was used to analyze and compare similarities with other proteins available in the database. A phylogenetic tree was constructed by aligning the nucleotide sequences of the genomes of LysPEF1-1, LysPEF1-2, and other homologous genes in the database using the neighbor-joining method with MEGA version 7.0 software. Bootstrap analysis with 1000 replicates was performed to assess the robustness of the phylogenetic tree. The percentages of replicate trees in which the associated taxa clustered together are indicated next to the branches of the tree. Three-dimensional structures of the endolysin proteins were prepared using the Phyre2 web portal and I-TASSER (https://zhanggroup.org/I-TASSER/; accessed on 21 August 2024) for protein modeling, prediction, and analysis, and the PyMOL program for drawing and marking.

### 4.3. Cloning, Overexpression, and Purification of LysPEF1-1 and LysPEF1-2

The LysPEF1-1 and LysPEF1-2 genes were amplified via polymerase chain reaction (PCR) using the primers pef1-1-Fw (5′-ATCAGCTAGCATGAACTACTCCCGAACAGG-3′), pef1-1-Rv (5′-ATCAGGATCCTTACTTAAATGTACCCCATGC-3′), pef1-2-Fw (5′-ATCAGCTAGCATGGCAGGAGAAGTATTTAG-3′), and pef1-2-Rv (5′-ATCAGGATCCTTACAATTTGTGAGTTCCACC-3′), which contained the NheI and BamHI restriction sites (underlined). The PCR products (873 and 1257 bp for LysPEF1-1 and LysPEF1-2, respectively) and vector pET28a (+) were purified using a PCR product extraction kit (Nippon Genetics Co., Ltd., Tokyo, Japan) and double-digested with NheI (New England Biolabs, UK) and BamHI (New England Biolabs, UK). The digested products were ligated using Ligation High Ver. 2.0 (TOYOBO, Osaka, Japan) according to the manufacturer’s instructions. The ligated products (pET28-lyspef1-1 and pET28-lyspef1-2) were transformed into *E. coli* BL21 (DE3) cells via electroporation (200 Ω, 1800 V, 25 μF). The transformant cells were incubated shortly and inoculated onto Luria Bertani Agar (LBA, Becton Dickinson, Sparks, MD, USA) plate containing 25 μg/mL of kanamycin (Nacalai Tesque, Inc., Kyoto, Japan) for positive plasmid selection. For expression, an overnight culture was inoculated in LB broth (25 μg/mL kanamycin) at a ratio of 1:100, and incubated at 37 °C, 120 rpm until an optical density of 0.6 at 600 nm (OD_600_). The transcription of LysPEF1-1 and LysPEF1-2 in *E. coli* BL21 (DE3) was induced by 1 mmol/L of isopropyl-β-d-thiogalactopyranoside (IPTG, Nacalai Tesque, Inc., Kyoto, Japan) at 16 °C for 20 h. The bacterial cells were harvested via centrifugation at 8000× *g* at 4 °C for 15 min. The pellets were resuspended in lysis buffer (50 mmol/L Na_2_HPO_4_-NaH_2_PO_4_ and 300 mmol/L NaCl) at a ratio of 1:10 (*w*/*v*) and sonicated using an ultrasonic disruptor model UP201 (Tomy Seiko Co., LTD, Tokyo, Japan) set at 30 s pulses at 30 s intervals. The cell debris was then removed via centrifugation at 12,000× *g* for 10 min at 4 °C. Proteins in the LysPEF1-1 and LysPEF1-2 supernatants were harvested via column chromatography using a His60 Ni Superflow Cartridge (Takara Bio Inc., Kusatsu, Japan). The purity of the proteins was confirmed using sodium dodecylsulfate-polyacrylamide gel electrophoresis (SDS-PAGE), and the concentrations were measured using NanoDrop™ One (NanoDrop Technologies LLC, Wilmington, DE, USA).

### 4.4. Lytic Activity Assay of Recombinant LysPEF1-1 and LysPEF1-2

The antimicrobial activities of LysPEF1-1 and LysPEF1-2 against *E. faecalis* JCM 7783^T^ were determined using the turbidity method described by Zhang et al. [48], with slight modifications. Briefly, overnight cell cultures were washed and suspended in 50 mmol/L Tris-HCl buffer (pH 8.0) to obtain an OD_600_ of 1.0. LysPEF1-1 and LysPEF1-2 preparations were 10-fold serially diluted with the Tris-HCl buffer, and 180 μL of fresh bacterial culture and 20 μL of endolysins were added to attain a final concentration ranging from 18.75 to 600 μg/mL. Tris-HCl buffer was used as the control. The mixture was incubated at 25 °C for 180 min, and the OD_600_ was measured every 5 min. 

### 4.5. Fluorescence and Time-Lapse Microscopies

Fluorescence microscopy was performed to confirm bacterial viability after endolysin treatment. The LIVE/DEAD BacLight Bacterial Viability Kit (Thermo Fisher Scientific Inc., Waltham, MA, USA), which uses a mixture of SYTO 9 and propidium iodide, was used according to the manufacturer’s instructions. Briefly, the cell suspension washed twice, resuspended in 1 mL 50 mmol/L Tris-HCl buffer (pH 8.0), mixed with a 3 μL dye mixture, and kept at 37 °C for 10 min. The bacterial cell membrane was monitored via fluorescence and time-lapse microscopy after staining with POLARIC solution (500 BCS; Nacalai Tesque, Kyoto, Japan) according to the manufacturer’s instructions. The prepared cell suspension after staining was treated with 150 μg/mL endolysin or Tris-HCl buffer as the control. The samples were immediately spotted onto glass slides, covered with a coverslip, and recorded every 3 min for 9 min for time-lapse microscopy. The first measurement was started 3 min after the addition of endolysin. Observations were made using an Olympus BX53 fluorescence microscope (Olympus, Tokyo, Japan) at 25 °C.

### 4.6. Characterization of Endolysin LysPEF1-1 and LysPEF1-2

The effects of pH on the lytic activity of the two endolysins were determined after being treated with buffers of different pH levels: phosphate-citrate (pH 2.0–7.0), Tris-HCl (pH 7.5–9.0), and carbonic acid-bicarbonate buffer (pH 9.0–12.0). Recombinant LysPEF1-1 and LysPEF1-2 were resuspended in 50 mmol/L of respective buffers and incubated at 25 °C for 24 h before evaluating their lytic activities. For the thermal stability test, the endolysins were incubated at −20, 4, 25, 37, 42, 50, 60, 70, 80, 90, and 100 °C for 1 h. To evaluate the effects of NaCl on lytic activities, the endolysins were resuspended in NaCl solutions with 0, 25, 50, 100, 500, 800, and 1000 mmol/L and incubated at 25 °C for 24 h. For the effects of metal ions on activities of endolysins, 4 mmol/L ethylenediamine-N, N, N′, N′-tetraacetic acid, disodium salt, and dihydrate (EDTA, Nacalai Tesque, Inc., Kyoto, Japan) was added into the endolysin solution to chelate metal ions and incubated at 25 °C for 1 h. EDTA-treated endolysins were mixed with an equal volume of Tris-HCl buffer containing 1 mmol/L metal ions (KCl, CaCl_2_, MgCl_2_, ZnSO_4_, FeSO_4_, CuCl_2_, or MnCl_2_). Lytic activity was assessed using the turbidity method, as mentioned above. 

Bacterial cells were harvested by centrifugation of the overnight preculture at 8000× *g* for 5 min and resuspended in 50 mmol/L Tris-HCl buffer (pH 8.0). Twenty microliters of the treated endolysin solution were added to 180 μL of *E. faecalis* suspension to attain a final concentration of 150 μg/mL, and the relative lytic activity was calculated after 1 h incubation at 25 °C according to the following equation. The untreated control consisted of recombinant proteins dissolved in 50 mmol/L Tris-HCl buffer (pH 8.0) and stored at 4 °C until lytic activity testing was conducted simultaneously with the treated samples.

Relative lytic activity (%) = 100 × [ΔOD_600_ of test (reaction with endolysin) − ΔOD_600_ of control (reaction with buffer)] (with treatment)/[ΔOD_600_ of test (reaction with endolysin) − ΔOD_600_ of control (reaction with buffer)] (untreated control).

### 4.7. Lytic Spectra of LysPEF1-1 and LysPEF1-2

The lytic spectra of LysPEF1-1 and LysPEF1-2 were determined against 84 strains, including 69 *Enterococcus* spp., 6 other gram-positive stains, and 9 g-negative strains. All bacterial suspensions were prepared by centrifuging the overnight cultures at 8000× *g* for 5 min and resuspending in 50 mmol/L Tris-HCl buffer (pH 8.0). Gram-negative bacteria were pretreated for 20 min with 50 mmol/L Tris-HCl buffer (pH 8.0) containing saturated chloroform. Then, 20 microliters of the treated endolysin solution were added to 180 μL of bacterial suspension to achieve a final concentration of 40 μg/mL. The lytic activity was assessed using the turbidity method described above and calculated after 2 h of incubation at 25 °C according to the following equation:

Lytic activity (%) = 100 × [ΔOD_600_ of test (reaction with endolysin) − ΔOD_600_ of control (reaction with buffer)]/initial OD_600_

To evaluate the lytic activity of the endolysins against different bacterial strains, lytic activity was categorized as follows: lytic activity greater than 50% was classified as strong; those less than 50% as weak; and those with less than 0% as no activity.

### 4.8. Effect of LysPEF1-1 and LysPEF1-2 on Viability of E. faecalis in Broth

The antimicrobial activities of LysPEF1-1 and LysPEF1-2 against *E. faecalis* E5 planktonic cells were determined by measuring viable counts as described previously [49], with slight modifications. Briefly, bacteria were grown overnight in brain-heart infusion (BHI) broth (Becton, Dickinson and Company, Franklin Lakes, NJ, USA) at 37 °C with shaking at 120 rpm. The cultures were then diluted 1:100 in fresh BHI broth to achieve a final cell concentration of approximately 10^6^ CFU/mL. Endolysins LysPEF1-1 and LysPEF1-2 were added separately or equally to attain a final concentration of 60 μg/mL. Equal volumes of 50 mmol/L Tris-HCl buffer (pH 8.0) or *E. faecalis* phage PEF1 at an MOI of 1 were used as controls. The mixtures were incubated at 4, 25, and 37 °C without shaking. After 2, 4, 6, 8, 12, 24, and 48 h of incubation, 200 μL samples were collected and serially diluted with Tris-HCl buffer. Dilutions were spotted (10 μL/spot) on TSA plates and incubated overnight at 37 °C. The viability of *E. faecalis* was calculated based on the number of colonies formed.

### 4.9. Effects of Endolysin LysPEF1-1 and LysPEF1-2 on Inhibiting E. faecalis Biofilm Formation

In a 96-well microplate, 180 μL of *E. faecalis* suspension (OD_600_ = 0.15) was either mixed with 20 μL of a single endolysin solution or with 10 μL each of two endolysin solutions to achieve a final enzyme concentration of 150 μg/mL for each treatment. The microplates were incubated at 4, 25, and 37 °C for 48 h. Biofilm mass was determined using the crystal violet staining method, as described previously [50]. Briefly, the suspension in the microplate was removed, and the wells were gently washed twice with sterile phosphate-buffered saline (PBS) to remove non-adherent cells, then dried in a biosafety cabinet. To each well, 200 μL of 1% crystal violet solution was added and kept at room temperature for 30 min. After removing the supernatant, each well was washed twice with sterile water and dried at room temperature. Subsequently, 200 μL of 99% ethanol was added to each well to dissolve the retained crystal violet. The biofilm mass was determined using a microplate reader (Infinite F NANO^+^, Tecan Group Ltd., Kawasaki, Japan) at an absorbance of 595 nm.

### 4.10. Treatment of E. faecalis Biofilms on 96-Well Microplate with LysPEF1-1 and LysPEF1-2

The effects of the two endolysins on biofilm cells were determined in 96-well plates according to the method of Miyamoto et al. [50] with modifications. Briefly, overnight cultures of *E. faecalis* JCM 7783^T^ were diluted to 10^6^ CFU/mL with fresh tryptic soy broth (TSB; Becton, Dickinson and Company) supplemented with 1.0% _D_-(+)-glucose. The cell suspensions were transferred to 96-well plates at 200 μL/well and incubated for 24 h at 37 °C without agitation. After biofilm formation, the wells were washed twice with PBS and resuspended in 200 μL of a single endolysin solution or an equal mixture of two endolysin solutions to attain the final concentration of 150 μg/mL. The microplates were incubated at 4, 25, or 37 °C for 2 h for the endolysin treatment. For the control, the same volume of 50 mmol/L Tris-HCl buffer (pH 8.0) was used. The supernatants were gently removed, and the wells were washed twice with Tris-HCl buffer. Biofilms were obtained by scraping the wells with a pipette tip and rinsing with Tris-HCl. The recovered cell suspension was vortexed for 30 s and serially diluted in the Tris-HCl buffer. Viable cell counts were determined by the plate count method. 

### 4.11. Treatment of E. faecalis Biofilm Cells on 304 Stainless Steel and Glass Surfaces

The efficacy of LysPEF1-1 and LysPEF1-2 was further determined on the surface of 304 stainless steel coupons (304 SSCs) and poly-L-lysine glass slides, which were presterilized at 121 °C for 20 min, and then dried at a 70 °C dryer. Biofilm formation and endolysin treatment methods were performed as described by Zhang et al. [51], with some modifications. Briefly, overnight cultures of *E. faecalis* JCM 7783^T^ were diluted in TSB supplemented with 1.0% _D_-(+)-glucose to obtain a concentration of 10^6^ CFU/mL. Then, 100 μL of the bacterial suspension was added onto the SSC surface or glass surface, placed in a sealed petri dish, and incubated at 37 °C for 24 h to allow biofilm formation. Subsequently, the cell suspension was removed, and the SSCs and glass slides were washed twice with PBS to remove planktonic cells. One hundred microliters of a single endolysin or an equal mixture of two endolysin solutions (150 μg/mL) was then added to the SSC surface or glass surfaces and incubated at 37 °C for 2 h. An equal volume of 50 mmol/L Tris-HCl buffer (pH 8.0) was used as the control. After the treatment, the SSCs and glass slides were washed twice with sterile water. To harvest biofilm cells, a sterile swab was used to swipe the surface of SSCs and glass slides, which were immersed in a sterilized tube containing 10 mL of 50 mmol/L Tris-HCl buffer (pH 8.0) and vortexed thoroughly. The biofilm cells were serially diluted in Tris-HCl buffer. Viable cell counts were determined by the plate count method. 

### 4.12. Statistical Analysis

All experiments were performed at least three times, with each experiment including multiple biological and technical replicates as appropriate. The data are presented as the mean ± standard deviation (SD). Statistical analysis was conducted using the SPSS 21.0 statistical analysis program. One-way analysis of variance (ANOVA) was used to determine the statistical significance of differences among groups. For comparisons between two groups, a two-tailed Student’s t-test was applied. For multiple comparisons, Tukey’s HSD was employed to identify significant differences between specific pairs of groups. A *p*-value < 0.05 was regarded as statistically significant. All replicates were treated independently, and no data points were excluded from the analysis.

## 5. Conclusions

To our knowledge, this is the first report on the antimicrobial effects of two endolysins from the same phage (PEF1) against MDR *E. faecalis* planktonic and biofilm cells. LysPEF1-1 and LysPEF1-2 showed rapid and strong lytic activity against *E. faecalis*, with LysPEF1-2 reducing the initial OD_600_ value to as low as 27% within 3 h. They exhibited the highest activity at 4 °C and 25 °C, tolerated NaCl concentrations ranging from 0 to 1000 mmol/L, and remained active after treatment with a pH range of 2–12 for LysPEF1-1 and 8–12 for LysPEF1-2. Both of them showed strong antimicrobial activity against MDR *E. faecalis* strains, which showed resistance to their parental phage PEF1. The combination of LysPEF1-1 and LysPEF1-2 showed an additional bactericidal effect on *E. faecalis* JCM 7783^T^, with a maximum 4.5 log reduction in viable count of biofilm cells at 4 °C on the glass slide surface. The strong antibacterial and antibiofilm activities of these endolysins suggest their potential as alternative agents against multi-drug resistant *E. faecalis* strains in the future.

## Figures and Tables

**Table 1:** The lytic spectrum of PEF1 and relative lytic activities of endolysins LysPEF1-1 and LysPEF1-2 on 69 *Enterococcus* spp.

Species	Strain Number	Phage PEF1	Lytic Activity (%)	
			LysPEF1-1	LysPEF1-2
*Enterococcus* *faecalis*	J1	±	66.32 ± 0.93	24.90 ± 4.06
	J2	±	49.21 ± 19.91	5.80 ± 3.51
	J3	±	69.96 ± 2.62	6.85 ± 2.79
	J4	±	39.80 ± 0.60	-
	J5	−	-	-
	J6	±	32.97 ± 6.70	-
	J7	±	56.83 ± 0.50	9.75 ± 1.16
	J8	+	78.46 ± 1.54	31.36 ± 1.36
	J9	−	62.44 ± 0.66	5.16 ± 0.96
	J10	±	73.74 ± 0.47	6.68 ± 0.02
	J11	+	70.10 ± 2.73	10.95 ± 0.64
	J12	±	13.84 ± 1.34	5.00 ± 2.71
	J13	±	53.28 ± 0.93	-
	J14	−	-	-
	J15	−	53.34 ± 0.76	-
	J16	±	46.97 ± 18.04	8.04 ± 5.13
	J17	−	67.62 ±5.55	1.41 ± 0.08
	J18	±	53.24 ± 0.32	8.15 ± 0.41
	J19	±	58.48 ± 14.42	8.58 ± 0.39
	J20	±	66.04 ± 12.23	8.85 ± 1.99
	J21	±	63.40 ± 7.80	20.13 ± 0.96
	J27	±	67.55 ± 1.81	38.62 ± 2.71
	J28	+	58.27 ± 15.35	70.66 ± 1.99
	J30	±	45.27 ± 2.70	60.67 ± 5.74
	J31	±	73.28 ± 0.42	33.75 ± 3.58
	J32	±	61.49 ± 2.14	12.89 ± 2.13
	J33	±	54.4 ± 0.86	20.69 ± 0.07
	J34	±	35.31 ± 0.37	24.65 ± 0.21
	JM9	±	62.00 ± 2.03	30.59 ± 0.89
	E1	±	60.87 ± 1.65	13.80 ± 1.76
	E2	±	38.98 ± 3.97	-
	E3	±	20.22 ± 2.00	-
	E4	±	68.55 ± 5.63	69.10 ± 2.12
	E5	−	57.65 ± 5.43	10.02 ± 0.76
	E6	±	46.52 ± 0.78	32.51 ± 5.01
	E7	±	61.84 ± 0.53	5.19 ± 0.91
	E8	±	64.48 ± 1.11	24.89 ± 0.50
	E9	±	10.41 ± 0.87	8.09 ± 1.78
	E10	±	78.70 ± 2.77	3.80 ± 0.19
	E11	±	25.21 ± 3.00	11.97 ± 0.56
	E12	±	73.37 ± 2.71	7.29 ± 1.29
	E13	±	74.89 ± 1.94	3.16 ± 1.78
	E14	+	75.12 ± 6.12	29.36 ± 0.93
	E15	±	59.98 ± 2.87	3.52 ± 0.58
	E16	±	15.80 ± 5.12	1.40 ± 0.76
	E17	±	80.98 ± 1.98	1.31 ± 0.11
	E18	±	69.40 ± 6.20	2.10 ± 0.94
	E19	±	58.77 ± 3.02	4.60 ± 0.32
	E20	±	70.49 ± 1.99	8.55 ± 1.85
	E21	±	73.08 ± 2.10	6.55 ± 2.63
	E22	±	77.95 ± 4.10	40.93 ± 5.12
	E23	±	69.36 ± 2.08	6.55 ± 1.81
	E24	±	41.74 ± 5.34	-
	E25	±	80.07 ± 3.58	20.09 ± 1.96
	E26	±	81.23 ± 2.13	28.73 ± 0.47
	E27	±	80.42 ± 1.36	23.29 ± 2.14
	E28	±	51.98 ± 3.10	19.40 ± 0.86
	E29	±	77.46 ± 5.85	15.36 ± 0.68
	E30	±	12.34 ± 0.88	2.99 ± 0.76
	JCM 7783^T^	±	65.34 ± 1.68	15.14 ± 1.29
	JCM 5803^T^	±	8.56 ± 1.70	11.18 ± 29.30
*Enterococcus* *faecium*	J22	−	-	14.47 ± 0.08
	J23	±	-	29.58 ± 0.45
	J24	±	-	20.83 ± 0.39
	J25	−	10.78 ± 0.82	5.16 ± 1.89
	J26	−	3.25 ± 0.87	12.22 ± 0.73
	J29	−	-	1.29 ± 0.71
	J35	−	-	-
	J36	±	3.71 ± 1.02	-

(+): Strong lysis, (±): Weak lysis, (−): Non-lysis or Lytic activity (%) < 0.

**Table 2:** The lytic spectrum of PEF1 and relative lytic activities of endolysins LysPEF1-1 and LysPEF1-2 on six gram-positive and nine gram-negative strains.

Species	Strain Number	Phage PEF1	Lytic Activity (%)	
			LysPEF1-1	LysPEF1-2
*Staphylococcus aureus*	NCTC 8325	−	-	2.20 ± 0.83
	No. 179	−	-	3.78 ± 1.64
*Listeria monocytogenes*	No. 185	−	23.59 ± 4.46	15.39 ± 1.39
*Bacillus cereus*	BC-RI15	−	-	2.85 ± 0.82
*Clostridium perfrigens*	JCM 1290^T^	−	16.25 ± 1.44	9.72 ± 1.79
	S1	−	16.77 ± 0.57	10.02 ± 1.14
*Escherichia coli*	O157:H7	−	-	-
*Salmonella* Typhimurium	IFO 12529	−	25.76 ± 7.42	26.35 ± 1.86
*S.* Enteritidis	IFO 3313	−	12.25 ± 0.99	10.84 ± 0.42
*Campylobacter jejuni*	L26	−	100.01 ± 0.22	101.51 ± 0.51
*C. coli*	Can 10	−	87.29 ± 0.17	103.91 ± 0.59
*Pseudomonas alcaligenes*	NBRC 14159	−	20.6 ± 1.00	19.24 ± 0.94
*P. fluorescens*	NBRC 14160	−	13.18 ± 2.70	19.34 ± 6.41
*P. fragi*	NBRC 3458	−	-	14.28 ± 0.63
*P. oleovorans*	NBRC 13583	−	9.74 ± 1.26	21.37 ± 0.80

(−): Non-lysis or Lytic activity (%) < 0.

## Data Availability

The data presented in this study are available upon request.

## Supplementary Materials

The following supporting information can be downloaded at: https://www.mdpi.com/article/10.3390/antibiotics13090884/s1. Figure S1: Genome map of phage PEF1-1. Green arrows: phage structure and packaging; Cyan arrows: DNA replication; Red arrows: host lysis; Red-purple arrows: hypothetical protein; Yellow arrows: additional functions. Gene bank accession number: OQ653963; Figure S2: Predicted secondary structure and disorder regions of endolysin LysPEF1-1 (**A**) and LysPEF1-2 (**B**). The structures of two endolysins were predicted using Phyre^2^. The predicted endolysin secondary structure (α-helices green, β-strands blue) and disordered regions were color-coded by confidence level; Figure S3: Expression of recombinant endolysins LysPEF1-1, LysPEF1-2. SDS-Page of LysPEF1-1 (**A**) and LysPEF1-2 (**B**); Figure S4: Effect on *E. faecalis* JCM 7783^T^ and E5 by endolysins LysPEF1-1 and LysPEF1-2. (**A**) The bacteria plaque was lysed with endolysin on TSA plate with LB molten-top soft agar; (**B**) The statue of E5 suspension after being treated by two endolysins in individual or mixing (with a concentration of 60 μg/mL) at 37 °C for 4 h incubation. The order from left to right: control group, LysPEF1-1, LysPEF1-2, combination of LysPEF1-1 and LysPEF1-2; Figure S5: Visualization of the control group *E. faecalis* JCM 7783^T^ cells in real-time lapse series. Exponentially growing cells (phase-contrast **A**) were stained by LIVE/DEAD™ Sperm Viability Kit (**B**) bacteria membrane detecting probe POLARIC-500BCS (**C**); Table S1: Bacterial strains used in this study; Table S2: Three-dimensional alignment results of LysPEF1-1 with similar protein templates. Table S3: Three-dimensional alignment results of LysPEF1-2 with similar protein templates.
