# Supplementary material for: Characterization of Two Novel Endolysins from Bacteriophage PEF1 and Evaluation of Their Combined Effects on the Control of Enterococcus faecalis Planktonic and Biofilm Cells
Source: Antibiotics (Basel). 2024 Sep 13;13(9):884. doi: 10.3390/antibiotics13090884 (PMC11428236; doi:10.3390/antibiotics13090884)
Supplement: Supplementary file 1 [file antibiotics-13-00884-s001.zip › 0821 Supplementary materials.docx]

**Figure S1** Genome map of phage PEF1-1. Green arrows: phage structure and packaging; Cyan arrows: DNA replication; Red arrows: host lysis; Red-purple arrows: hypothetical protein; Yellow arrows: additional functions. Gene bank accession number: OQ653963.

**
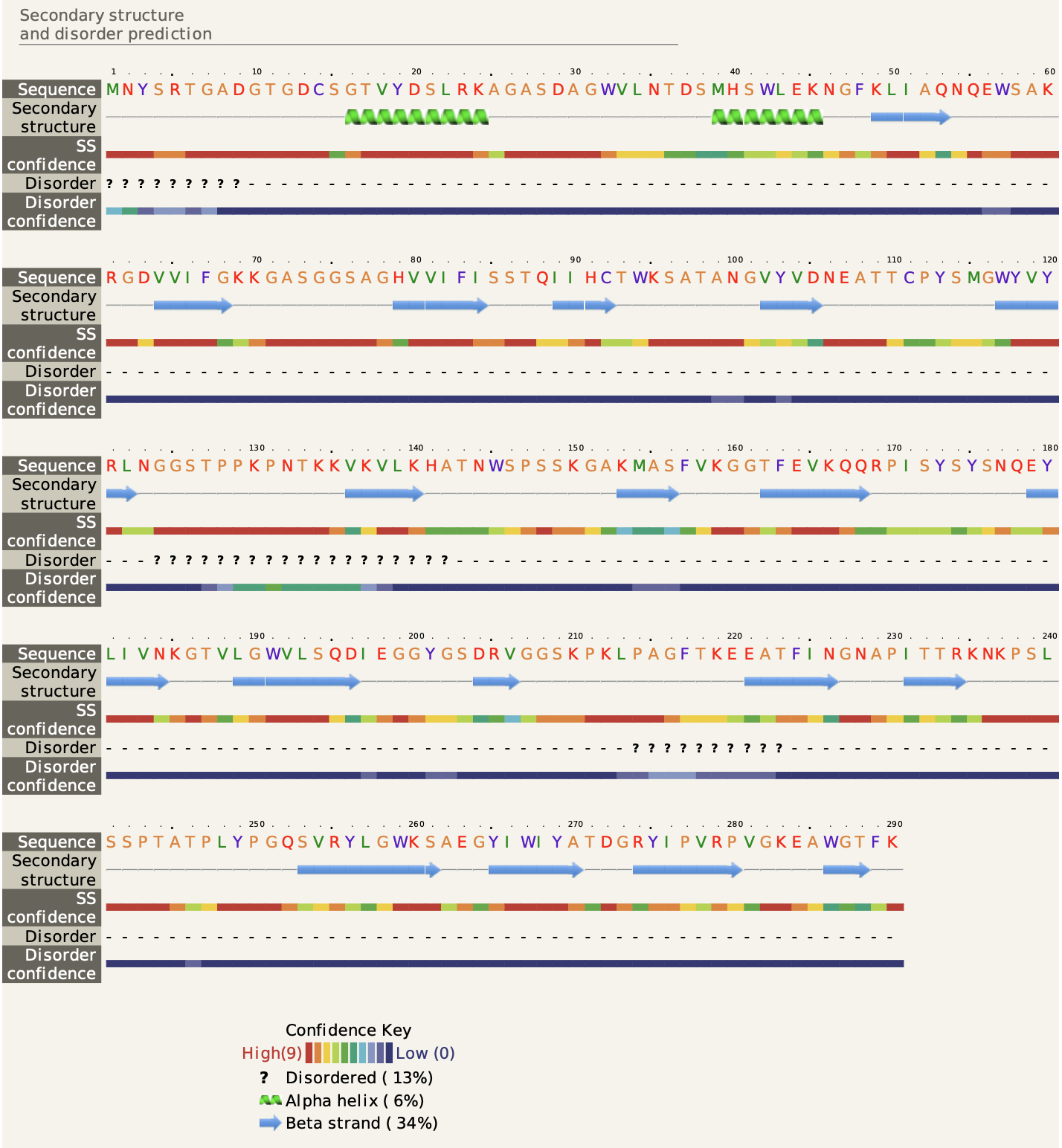
(A)**

**(B)**

**Figure S2** Predicted secondary structure and disorder regions of endolysin LysPEF1-1 (A) and LysPEF1-2 (B). The structures of two endolysins were predicted using Phyre^2^. The predicted endolysin secondary structure (α-helices green, β-strands blue) and disordered regions were color-coded by confidence level.

**(A)**

** (B)**

**Figure S3** Expression of recombinant endolysins LysPEF1-1, LysPEF1-2. SDS-Page of LysPEF1-1 (A) and LysPEF1-2 (B).

**(A)**


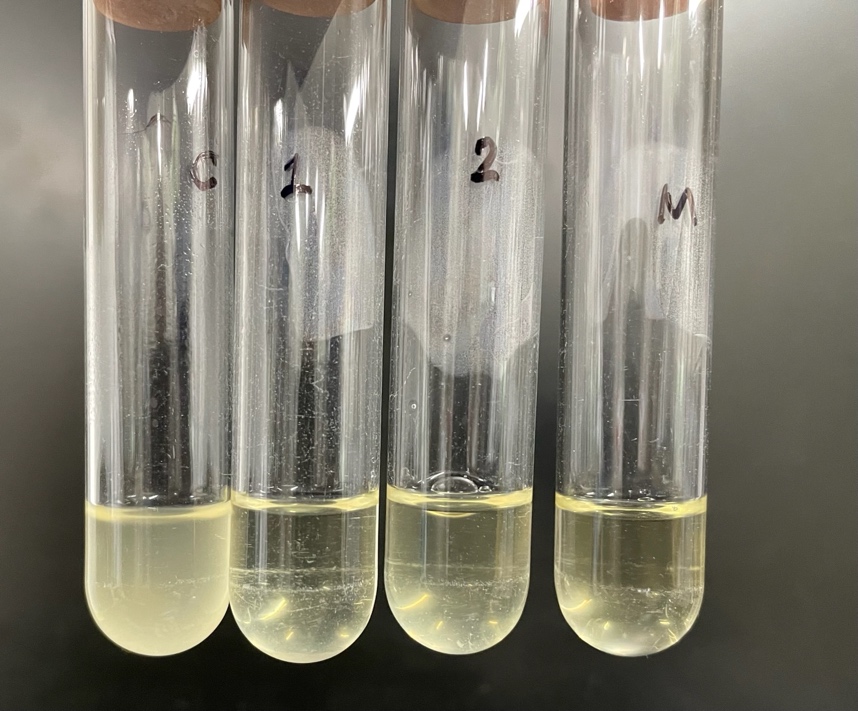


**(B)**

**Figure S4** Effect on *E. faecalis* JCM 7783^T^ and E5 by endolysins LysPEF1-1 and LysPEF1-2. (A) The bacteria plaque was lysed with endolysin on TSA plate with LB molten-top soft agar; (B) The statue of E5 suspension after being treated by two endolysins in individual or mixing (with a concentration of 60 μg/mL) at 37 °C for 4 h incubation. The order from left to right: control group, LysPEF1-1, LysPEF1-2, combination of LysPEF1-1 and LysPEF1-2.

**Figure S5** Visualization of the control group *E. faecalis* JCM7783^T^ cells in real-time lapse series. Exponentially growing cells (phase-contrast A) were stained by LIVE/DEAD™ Sperm Viability Kit (B) bacteria membrane detecting probe POLARIC -500BCS (C). It was dropped onto a poly-L-lysine glass slide and covered with a coverslip and monitored. Three-minute intervals are shown for 3, 6, and 9 min. The live cell showed as green color and the dead cell with red color. White arrows represent live cells. Scale bar=10 µm

**Table S1** Bacterial strains used in this study

| **Strain** | **Number** | **Source** |
| --- | --- | --- |
| *Enterococcus faecalis* | J1, J2, J3, J4, J5, J6, J7, J8, J9, J10, J11, J12, J13, J14, J15, J16, J17, J18, J19, J20, J21, J27, J28, J30, J31, J32, J33, J34, JM9 | Isolated by FHC from food in Fukuoka, Japan |
|  | E1, E2, E3, E4, E5, E6, E7, E8, E9, E10, E11, E12, E13, E14, E15, E16, E17, E18, E19, E20, E21, E22, E23, E24, E25, E26, E27, E28, E29, E30 | Isolated by FHC from food in Cairo, Zagazig, Giza of Egypt |
|  | JCM 7783^T^, JCM 5803^T^ | Japan Collection of Microorganisms |
| *Enterococcus faecium* | J22, J23, J24, J25, J26, J29 | Isolated by FHC from food in Fukuoka, Japan |
| *Enterococcus* *casseliflavus* | J35 |  |
| *E**nterococcus gallinarum* | J36 |  |
| *Escherichia coli* | BL21(DE3) | New England Biolabs (UK) |
| *Staphylococcus aureus* | NCTC 8325 | National Collection of Type Cultures  of Public Health England, Salisbury, SP4 0JG UK |
|  | No. 179 | Isolated by FHC from food in Fukuoka, Japan |
| *Listeria monocytogenes* | No. 185 | Public Health Center,  Saku, Nagano, Japan |
| *Bacillus cereus* | BC-RI15 | Isolated by FHC from food in Fukuoka, Japan |
| *Clostridium perfrigens* | JCM1290^T^ | Japan Collection of Microorganisms |
|  | S1 |  |
| *Escherichia coli* | O157:H7 | Fukuoka City Institute of Health and Environment, Japan |
| *Salmonella* Typhimurium | IFO 12529 | Biological Resource Center, National Institute of Technology and Evaluation (NBRC), Chiba, Japan |
| *Salmonella* Enteritidis | IFO 3313 | Isolated by FHC from food in Fukuoka, Japan |
| Campylobacter jejuni | L26 |  |
| Campylobacter coli | Can 10 |  |
| *Pseudomonas alcaligenes* | NBRC 14159 | Biological Resource Center, National Institute of Technology and Evaluation (NBRC), Chiba, Japan |
| *Pseudomonas fluorescens* | NBRC 14160 |  |
| *Pseudomonas fragi* | NBRC 3458 |  |
| *Pseudomonas oleovorans* | NBRC13583 |  |
