# Supplementary material for: Characterization of Two Novel Endolysins from Bacteriophage PEF1 and Evaluation of Their Combined Effects on the Control of Enterococcus faecalis Planktonic and Biofilm Cells
Source: Antibiotics (Basel). 2024 Sep 13;13(9):884. doi: 10.3390/antibiotics13090884 (PMC11428236; doi:10.3390/antibiotics13090884)
Supplement: Supplementary file 1 [file antibiotics-13-00884-s001.zip › Table S2.pdf]

Table S2: Three-dimensional alignment results of LysPEF1-1 with similar protein templates.

Phyre2

Email	wangchen950518@gmail.com
Description	LysPEF-1_290_aa_
Date	Wed Aug 21 04:14:06 BST 2024
Unique Job ID	69ab052c5c7c03e2

Detailed template information

#	Template	Alignment Coverage	3D Model	Confidence	% i.d.	Template Information
1	c8h1iA_	Alignment	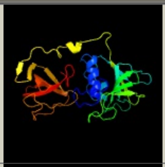	99.6	23	**PDB header:** antimicrobial protein<br>**Chain:** A: **PDB Molecule:** endolysin;<br>**PDBTitle:** crystal structure of plygrcs, a bacteriophage endolysin in complex2 with cold shock protein c<br>**PDB Entry:** PDBe RCSB PDBj
2	c5udmA_	Alignment	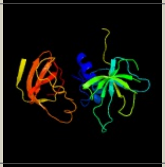	99.4	26	**PDB header:** hydrolase<br>**Chain:** A: **PDB Molecule:** phage-associated cell wall hydrolase;<br>**PDBTitle:** phage-associated cell wall hydrolase plypy from streptococcus2 pyogenes, space group p6522<br>**PDB Entry:** PDBe RCSB PDBj
3	c8tw1A_	Alignment	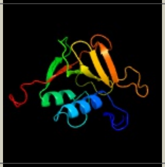	100.0	31	**PDB header:** hydrolase<br>**Chain:** A: **PDB Molecule:** endolysin lys2972;<br>**PDBTitle:** crystal structure of lys2972, a phage endolysin targeting2 streptococcus thermophilus<br>**PDB Entry:** PDBe RCSB PDBj
4	c2mk5A_	Alignment	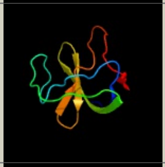	99.4	46	**PDB header:** hydrolase<br>**Chain:** A: **PDB Molecule:** endolysin;<br>**PDBTitle:** solution structure of a protein domain<br>**PDB Entry:** PDBe RCSB PDBj
5	c4f0wA_	Alignment	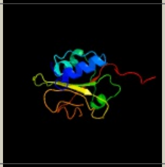	93.8	28	**PDB header:** hydrolase<br>**Chain:** A: **PDB Molecule:** putative uncharacterized protein;<br>**PDBTitle:** crystal structure of type effector tse1 c30a mutant from pseudomonas2 aeruginousa<br>**PDB Entry:** PDBe RCSB PDBj
6	c4lxcA_	Alignment	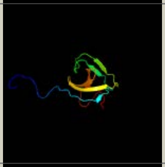	98.1	34	**PDB header:** hydrolase<br>**Chain:** A: **PDB Molecule:** lysostaphin;<br>**PDBTitle:** the antimicrobial peptidase lysostaphin from staphylococcus simulans<br>**PDB Entry:** PDBe RCSB PDBj
7	c2xivA_	Alignment	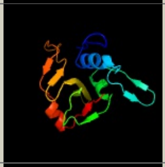	99.8	29	**PDB header:** structural protein<br>**Chain:** A: **PDB Molecule:** hypothetical invasion protein;<br>**PDBTitle:** structure of rv1477, hypothetical invasion protein of mycobacterium2 tuberculosis<br>**PDB Entry:** PDBe RCSB PDBj
8	c8ev5A_	Alignment	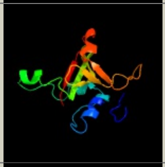	99.7	28	**PDB header:** hydrolase/inhibitor<br>**Chain:** A: **PDB Molecule:** clan ca, family c40, nlpc/p60 superfamily cysteine<br>**PDBTitle:** nlpc b3 covalently bound with e64 inhibitor fragment<br>**PDB Entry:** PDBe RCSB PDBj
9	c2k1gA_	Alignment	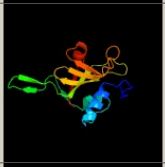	99.7	26	**PDB header:** lipoprotein<br>**Chain:** A: **PDB Molecule:** lipoprotein spr;<br>**PDBTitle:** solution nmr structure of lipoprotein spr from escherichia coli k12.2 northeast structural genomics target er541-37-162<br>**PDB Entry:** PDBe RCSB PDBj
10	c1r77A_	Alignment	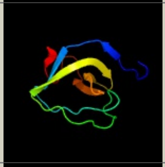	99.2	32	**PDB header:** hydrolase<br>**Chain:** A: **PDB Molecule:** cell wall targeting domain of glycylglycine endopeptidase<br>**PDBTitle:** crystal structure of the cell wall targeting domain of peptidylglycan2 hydrolase ale-1<br>**PDB Entry:** PDBe RCSB PDBj
11	c7cflB_	Alignment	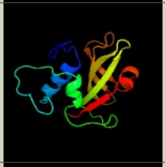	99.7	25	**PDB header:** hydrolase<br>**Chain:** B: **PDB Molecule:** putative cell wall hydrolase phosphatase-associated<br>**PDBTitle:** x-ray structure of autolysin acd24020 catalytic domain from2 clostridium difficile<br>**PDB Entry:** PDBe RCSB PDBj
			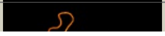			

12	c3npfB_	Alignment	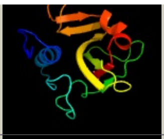	99.8	24	**PDB header:** hydrolase<br>**Chain:** B: **PDB Molecule:** putative dipeptidyl-peptidase vi;<br>**PDBTitle:** crystal structure of a putative dipeptidyl-peptidase vi (bacova_00612)2 from bacteroides ovatus at 1.72 a resolution<br>**PDB Entry:** PDBe RCSB PDBj
13	c8wt3A_	Alignment	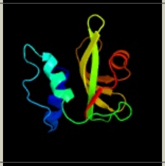	99.7	25	**PDB header:** hydrolase<br>**Chain:** A: **PDB Molecule:** peptidoglycan dl-endopeptidase cwlo;<br>**PDBTitle:** crystal structure of peptidoglycan dl-endopeptidase cwlo<br>**PDB Entry:** PDBe RCSB PDBj
14	d2evra2	Alignment	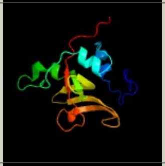	99.9	21	**Fold:** Cysteine proteinases<br>**Superfamily:** Cysteine proteinases<br>**Family:** NlpC/P60<br>**PDB entry:** PDBe RCSB PDBj
15	c8i2eD_	Alignment	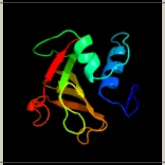	99.7	24	**PDB header:** antimicrobial protein/inhibitor<br>**Chain:** D: **PDB Molecule:** probable peptidoglycan endopeptidase lyte;<br>**PDBTitle:** crystal structure of bacillus subtilis lyte in complex with isea<br>**PDB Entry:** PDBe RCSB PDBj
16	c8er5B_	Alignment	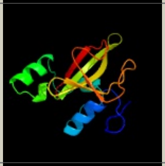	99.7	24	**PDB header:** hydrolase<br>**Chain:** B: **PDB Molecule:** nlpc/p60 domain-containing protein;<br>**PDBTitle:** crystal structure of nlpc/p60 domain from clostridium innocuum2 nlpc/p60 domain-containing protein ci_01448.<br>**PDB Entry:** PDBe RCSB PDBj
17	c7aqhH_	Alignment	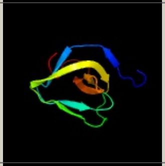	98.9	29	**PDB header:** viral protein<br>**Chain:** H: **PDB Molecule:** orf007;<br>**PDBTitle:** cell wall binding domain of the staphylococcal phage 2638a endolysin<br>**PDB Entry:** PDBe RCSB PDBj
18	c6b8cA_	Alignment	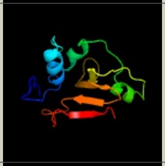	99.7	26	**PDB header:** hydrolase<br>**Chain:** A: **PDB Molecule:** nlp/p60;<br>**PDBTitle:** crystal structure of nlpc/p60 domain of peptidoglycan hydrolase saga<br>**PDB Entry:** PDBe RCSB PDBj
19	c3pbiA_	Alignment	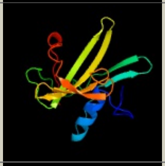	99.8	23	**PDB header:** hydrolase<br>**Chain:** A: **PDB Molecule:** invasion protein;<br>**PDBTitle:** structure of the peptidoglycan hydrolase ripb (rv1478) from2 mycobacterium tuberculosis at 1.6 resolution<br>**PDB Entry:** PDBe RCSB PDBj
20	c3h41A_	Alignment	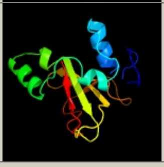	99.7	21	**PDB header:** hydrolase<br>**Chain:** A: **PDB Molecule:** nlp/p60 family protein;<br>**PDBTitle:** crystal structure of a nlpc/p60 family protein (bce_2878) from2 bacillus cereus atcc 10987 at 1.79 a resolution<br>**PDB Entry:** PDBe RCSB PDBj
21	c4hpeA_	Alignment	not modelled	99.8	21	**PDB header:** hydrolase<br>**Chain:** A: **PDB Molecule:** putative cell wall hydrolase tn916-like,ctn1-orf17;<br>**PDBTitle:** crystal structure of a putative cell wall hydrolase (cd630_03720) from2 clostridium difficile 630 at 2.38 a resolution<br>**PDB Entry:** PDBe RCSB PDBj
22	c8jiaE_	Alignment	not modelled	99.9	23	**PDB header:** transport protein<br>**Chain:** E: **PDB Molecule:** probable endopeptidase mt2245;<br>**PDBTitle:** cryo-em structure of mycobacterium tuberculosis atp bound ftse(e165q)2 x/ripc complex in peptidisc<br>**PDB Entry:** PDBe RCSB PDBj
23	c7bk8A_	Alignment	not modelled	98.1	27	**PDB header:** hydrolase<br>**Chain:** A: **PDB Molecule:** magc;<br>**PDBTitle:** x-ray crystal structure of pseudomonas aeruginosa magc<br>**PDB Entry:** PDBe RCSB PDBj
24	c2fg0B_	Alignment	not modelled	99.9	23	**PDB header:** hydrolase<br>**Chain:** B: **PDB Molecule:** cog0791: cell wall-associated hydrolases (invasion-<br>**PDBTitle:** crystal structure of a putative gamma-d-glutamyl-l-diamino acid2 endopeptidase (npun_r0659) from nostoc punctiforme pcc 73102 at 1.793 a resolution<br>**PDB Entry:** PDBe RCSB PDBj
25	c4olkB_	Alignment	not modelled	97.9	20	**PDB header:** hydrolase<br>**Chain:** B: **PDB Molecule:** endolysin;<br>**PDBTitle:** the chap domain of lysgh15<br>**PDB Entry:** PDBe RCSB PDBj
26	c4f4mA_	Alignment	not modelled	93.2	30	**PDB header:** hydrolase regulator<br>**Chain:** A: **PDB Molecule:** papain peptidoglycan amidase effector tse1;<br>**PDBTitle:** structure of the type vi peptidoglycan amidase effector tse1 (c30a)2 from pseudomonas aeruginosa<br>**PDB Entry:** PDBe RCSB PDBj
						**PDB header:** hydrolase

27	c6istC_	Alignment	not modelled	98.1	27	**Chain:** C: **PDB Molecule:** lysin;<br>**PDBTitle:** crystal structure of a wild type endolysin lysime-ef1<br>**PDB Entry:** PDBe RCSB PDBj
28	c8aucB_	Alignment	not modelled	99.9	20	**PDB header:** cell cycle<br>**Chain:** B: **PDB Molecule:** cell wall-associated hydrolases (invasion-associated<br>**PDBTitle:** structure of peptidoglycan hydrolase cg1735 from corynebacterium2 glutamicum, trigonal crystal form<br>**PDB Entry:** PDBe RCSB PDBj
29	c8audB_	Alignment	not modelled	99.9	20	**PDB header:** cell cycle<br>**Chain:** B: **PDB Molecule:** cell wall-associated hydrolases (invasion-associated<br>**PDBTitle:** structure of peptidoglycan hydrolase cg1735 from corynebacterium2 glutamicum, orthorhombic crystal form<br>**PDB Entry:** PDBe RCSB PDBj
30	c6biqA_	Alignment	not modelled	99.9	18	**PDB header:** hydrolase<br>**Chain:** A: **PDB Molecule:** clan ca, family c40, nlpc/p60 superfamily cysteine<br>**PDBTitle:** structure of nlpc2 from trichomonas vaginalis<br>**PDB Entry:** PDBe RCSB PDBj
31	c4xcmB_	Alignment	not modelled	99.6	22	**PDB header:** hydrolase<br>**Chain:** B: **PDB Molecule:** cell wall-binding endopeptidase-related protein;<br>**PDBTitle:** crystal structure of the putative nlpc/p60 d,l endopeptidase from t.2 thermophilus<br>**PDB Entry:** PDBe RCSB PDBj
32	c5d74B_	Alignment	not modelled	98.4	23	**PDB header:** hydrolase<br>**Chain:** B: **PDB Molecule:** putative phage lysin;<br>**PDBTitle:** the crystal structure of ly7917<br>**PDB Entry:** PDBe RCSB PDBj
33	c4fdyA_	Alignment	not modelled	99.8	17	**PDB header:** hydrolase<br>**Chain:** A: **PDB Molecule:** similar to lipoprotein, nlp/p60 family;<br>**PDBTitle:** crystal structure of a similar to lipoprotein, nlp/p60 family2 (sav0400) from staphylococcus aureus subsp. aureus mu50 at 2.23 a3 resolution<br>**PDB Entry:** PDBe RCSB PDBj
34	c4hfkC_	Alignment	not modelled	38.4	19	**PDB header:** hydrolase<br>**Chain:** C: **PDB Molecule:** putative cytoplasmic protein;<br>**PDBTitle:** crystal structure of the type vi effector-immunity complex tae4-tai42 from enterobacter cloacae<br>**PDB Entry:** PDBe RCSB PDBj
35	c4hffA_	Alignment	not modelled	58.7	21	**PDB header:** hydrolase<br>**Chain:** A: **PDB Molecule:** putative cytoplasmic protein;<br>**PDBTitle:** crystal structure of the type vi effector-immunity complex tae4-tai42 from salmonella typhimurium<br>**PDB Entry:** PDBe RCSB PDBj
36	c4hzbA_	Alignment	not modelled	98.0	24	**PDB header:** hydrolase<br>**Chain:** A: **PDB Molecule:** putative cytoplasmic protein;<br>**PDBTitle:** crystal structure of the type vi semet effector-immunity complex tae3-2 tai3 from ralstonia pickettii<br>**PDB Entry:** PDBe RCSB PDBj
37	c3i86A_	Alignment	not modelled	99.7	18	**PDB header:** hydrolase<br>**Chain:** A: **PDB Molecule:** putative uncharacterized protein;<br>**PDBTitle:** crystal structure of the p60 domain from m. avium subspecies2 paratuberculosis antigen map1204<br>**PDB Entry:** PDBe RCSB PDBj
38	c2jtcA_	Alignment	not modelled	25.7	19	**PDB header:** hydrolase<br>**Chain:** A: **PDB Molecule:** streptopain;<br>**PDBTitle:** 3d structure and backbone dynamics of spe b<br>**PDB Entry:** PDBe RCSB PDBj
39	c3m1uB_	Alignment	not modelled	98.6	21	**PDB header:** hydrolase<br>**Chain:** B: **PDB Molecule:** putative gamma-d-glutamyl-l-diamino acid endopeptidase;<br>**PDBTitle:** crystal structure of a putative gamma-d-glutamyl-l-diamino acid2 endopeptidase (dvu_0896) from desulfovibrio vulgaris hildenborough at3 1.75 a resolution<br>**PDB Entry:** PDBe RCSB PDBj
40	c4emeB_	Alignment	not modelled	45.5	19	**PDB header:** hydrolase<br>**Chain:** B: **PDB Molecule:** m18 aspartyl aminopeptidase;<br>**PDBTitle:** x-ray crystal structure and specificity of the plasmodium falciparum2 malaria aminopeptidase<br>**PDB Entry:** PDBe RCSB PDBj
41	c6sqxA_	Alignment	not modelled	98.6	20	**PDB header:** hydrolase<br>**Chain:** A: **PDB Molecule:** peptide-binding protein;<br>**PDBTitle:** insights into a novel nlpc/p60 endopeptidase from photobacterium2 damselae subsp. piscicida<br>**PDB Entry:** PDBe RCSB PDBj
42	c3bb7A_	Alignment	not modelled	21.9	18	**PDB header:** hydrolase<br>**Chain:** A: **PDB Molecule:** interpain a;<br>**PDBTitle:** structure of prevotella intermedia prointerpain a fragment 39-3592 (mutant c154a)<br>**PDB Entry:** PDBe RCSB PDBj
43	c2lieA_	Alignment	not modelled	30.5	21	**PDB header:** sugar binding protein<br>**Chain:** A: **PDB Molecule:** ccl2 lectin;<br>**PDBTitle:** nmr structure of the lectin ccl2<br>**PDB Entry:** PDBe RCSB PDBj
44	c3gt2A_	Alignment	not modelled	99.7	16	**PDB header:** unknown function<br>**Chain:** A: **PDB Molecule:** putative uncharacterized protein;<br>**PDBTitle:** crystal structure of the p60 domain from m. avium paratuberculosis2 antigen map1272c<br>**PDB Entry:** PDBe RCSB PDBj
45	c4xaaA_	Alignment	not modelled	53.8	31	**PDB header:** oxidoreductase<br>**Chain:** A: **PDB Molecule:** putative oxygenase;<br>**PDBTitle:** crystal structure of avio1 from streptomyces viridochromogenes tue57<br>**PDB Entry:** PDBe RCSB PDBj
46	c4h4jA_	Alignment	not modelled	74.4	18	**PDB header:** structural genomics, unknown function<br>**Chain:** A: **PDB Molecule:** hypothetical protein;<br>**PDBTitle:** crystal structure of a n-acetylmuramoyl-l-alanine amidase2 (bacuni_02947) from bacteroides uniformis atcc 8492 at 1.15 a3 resolution<br>**PDB Entry:** PDBe RCSB PDBj
						**PDB header:** hydrolase

47	c5t1qB_	Alignment	not modelled	93.8	20	**Chain:** B: **PDB Molecule:** n-acetylmuramoyl-l-alanine amidase domain-containing<br>**PDBTitle:** 2.15 angstrom crystal structure of n-acetylmuramoyl-l-alanine amidase2 from staphylococcus aureus.<br>**PDB Entry:** PDBe RCSB PDBj
48	c8b2sA_	Alignment	not modelled	93.2	16	**PDB header:** hydrolase<br>**Chain:** A: **PDB Molecule:** gh24 family muramidase;<br>**PDBTitle:** gh24 family muramidase from trichophaea saccata with an sh3-like cell2 wall binding domain<br>**PDB Entry:** PDBe RCSB PDBj
49	c2p1gA_	Alignment	not modelled	98.4	18	**PDB header:** structural genomics, unknown function<br>**Chain:** A: **PDB Molecule:** putative xylanase;<br>**PDBTitle:** crystal structure of a putative xylanase from bacteroides fragilis<br>**PDB Entry:** PDBe RCSB PDBj
50	d1b12a_	Alignment	not modelled	38.4	35	**Fold:** LexA/Signal peptidase<br>**Superfamily:** LexA/Signal peptidase<br>**Family:** Type 1 signal peptidase<br>**PDB entry:** PDBe RCSB PDBj
51	c4cgkA_	Alignment	not modelled	88.5	20	**PDB header:** cell cycle<br>**Chain:** A: **PDB Molecule:** secreted 45 kda protein;<br>**PDBTitle:** crystal structure of the essential protein pcsb from streptococcus2 pneumoniae<br>**PDB Entry:** PDBe RCSB PDBj
52	c2lrjA_	Alignment	not modelled	96.4	21	**PDB header:** biosynthetic protein<br>**Chain:** A: **PDB Molecule:** staphyloxanthin biosynthesis protein, putative;<br>**PDBTitle:** nmr solution structure of staphyloxanthin biosynthesis protein<br>**PDB Entry:** PDBe RCSB PDBj
53	c4lt5A_	Alignment	not modelled	39.8	24	**PDB header:** oxidoreductase/dna<br>**Chain:** A: **PDB Molecule:** naegleria tet-like dioxygenase;<br>**PDBTitle:** structure of a naegleria tet-like dioxygenase in complex with 5-2 methylcytosine dna<br>**PDB Entry:** PDBe RCSB PDBj
54	c4wviA_	Alignment	not modelled	60.4	22	**PDB header:** hydrolase<br>**Chain:** A: **PDB Molecule:** maltose-binding periplasmic protein,signal peptidase ib;<br>**PDBTitle:** crystal structure of the type-i signal peptidase from staphylococcus2 aureus (spsb) in complex with a substrate peptide (pep2).<br>**PDB Entry:** PDBe RCSB PDBj
55	c7p2qA_	Alignment	not modelled	75.8	15	**PDB header:** membrane protein<br>**Chain:** A: **PDB Molecule:** signal peptidase complex catalytic subunit sec11c;<br>**PDBTitle:** human signal peptidase complex paralog c (spc-c)<br>**PDB Entry:** PDBe RCSB PDBj
56	c2k3aA_	Alignment	not modelled	95.6	20	**PDB header:** hydrolase<br>**Chain:** A: **PDB Molecule:** chap domain protein;<br>**PDBTitle:** nmr solution structure of staphylococcus saprophyticus chap (cysteine,2 histidine-dependent amidohydrolases/peptidases) domain protein.3 northeast structural genomics consortium target syr11<br>**PDB Entry:** PDBe RCSB PDBj
57	d1zcea1	Alignment	not modelled	84.5	21	**Fold:** PUA domain-like<br>**Superfamily:** PUA domain-like<br>**Family:** Atu2648/PH1033-like<br>**PDB entry:** PDBe RCSB PDBj
58	d2gbsa1	Alignment	not modelled	79.8	21	**Fold:** PUA domain-like<br>**Superfamily:** PUA domain-like<br>**Family:** Atu2648/PH1033-like<br>**PDB entry:** PDBe RCSB PDBj
59	c3kw0D_	Alignment	not modelled	96.6	19	**PDB header:** hydrolase<br>**Chain:** D: **PDB Molecule:** cysteine peptidase;<br>**PDBTitle:** crystal structure of cysteine peptidase (np_982244.1) from bacillus2 cereus atcc 10987 at 2.50 a resolution<br>**PDB Entry:** PDBe RCSB PDBj
60	c8ej5A_	Alignment	not modelled	32.5	12	**PDB header:** virus<br>**Chain:** A: **PDB Molecule:** gp10, tail tip lysin (spike);<br>**PDBTitle:** tail tip structure of staphylococcus phage andhra<br>**PDB Entry:** PDBe RCSB PDBj
61	c3kopB_	Alignment	not modelled	50.8	25	**PDB header:** structural genomics, unknown function<br>**Chain:** B: **PDB Molecule:** uncharacterized protein;<br>**PDBTitle:** crystal structure of protein with a cyclophilin-like fold2 (yp_831253.1) from arthrobacter sp. fb24 at 1.90 a resolution<br>**PDB Entry:** PDBe RCSB PDBj
62	d2im9a1	Alignment	not modelled	36.9	17	**Fold:** Cysteine proteinases<br>**Superfamily:** Cysteine proteinases<br>**Family:** Lpg0564-like<br>**PDB entry:** PDBe RCSB PDBj
63	c2im9A_	Alignment	not modelled	36.9	17	**PDB header:** structural genomics, unknown function<br>**Chain:** A: **PDB Molecule:** hypothetical protein;<br>**PDBTitle:** crystal structure of protein lpg0564 from legionella pneumophila str.2 philadelphia 1, pfam duf1460<br>**PDB Entry:** PDBe RCSB PDBj
64	d2io8a2	Alignment	not modelled	94.5	22	**Fold:** Cysteine proteinases<br>**Superfamily:** Cysteine proteinases<br>**Family:** CHAP domain<br>**PDB entry:** PDBe RCSB PDBj
65	c2ioaA_	Alignment	not modelled	87.1	22	**PDB header:** ligase, hydrolase<br>**Chain:** A: **PDB Molecule:** bifunctional glutathionylspermidine synthetase/amidase;<br>**PDBTitle:** e. coli bifunctional glutathionylspermidine synthetase/amidase2 incomplex with mg2+ and adp and phosphinate inhibitor<br>**PDB Entry:** PDBe RCSB PDBj
66	c5m0tA_	Alignment	not modelled	42.7	28	**PDB header:** oxidoreductase<br>**Chain:** A: **PDB Molecule:** alpha-ketoglutarate-dependent non-heme iron oxygenase eash;<br>**PDBTitle:** alpha-ketoglutarate-dependent non-heme iron oxygenase eash<br>**PDB Entry:** PDBe RCSB PDBj
						**PDB header:** oxidoreductase

67	c5daqA_	Alignment	not modelled	26.7	28	**Chain:** A: **PDB Molecule:** phytanoyl-coa dioxygenase family protein (afu_orthologue<br>**PDBTitle:** fe(ii)/(alpha)ketoglutarate-dependent dioxygenase asqj in complex with2 4-methoxycyclopeptin<br>**PDB Entry:** PDBe RCSB PDBj
68	d2a1xa1	Alignment	not modelled	44.2	32	**Fold:** Double-stranded beta-helix<br>**Superfamily:** Clavaminate synthase-like<br>**Family:** PhyH-like<br>**PDB entry:** PDBe RCSB PDBj
69	c5ncjB_	Alignment	not modelled	37.2	32	**PDB header:** oxidoreductase<br>**Chain:** B: **PDB Molecule:** leucine hydroxylase;<br>**PDBTitle:** grie in complex with manganese, succinate and (2s,4r)-5-hydroxyleucine<br>**PDB Entry:** PDBe RCSB PDBj
70	c3eopB_	Alignment	not modelled	77.0	17	**PDB header:** unknown function<br>**Chain:** B: **PDB Molecule:** thymocyte nuclear protein 1;<br>**PDBTitle:** crystal structure of the duf55 domain of human thymocyte nuclear2 protein 1<br>**PDB Entry:** PDBe RCSB PDBj
71	c2rdsA_	Alignment	not modelled	47.4	15	**PDB header:** oxidoreductase<br>**Chain:** A: **PDB Molecule:** 1-deoxypentalenic acid 11-beta hydroxylase; fe(ii)/alpha-<br>**PDBTitle:** crystal structure of ptlh with fe/oxalylglycine and ent-1-2 deoxypentalenic acid bound<br>**PDB Entry:** PDBe RCSB PDBj
72	c4n31A_	Alignment	not modelled	22.0	24	**PDB header:** cell adhesion<br>**Chain:** A: **PDB Molecule:** sipa;<br>**PDBTitle:** structure and activity of streptococcus pyogenes sipa: a signal2 peptidase homologue essential for pilus polymerisation<br>**PDB Entry:** PDBe RCSB PDBj
73	c8b2gB_	Alignment	not modelled	48.4	12	**PDB header:** hydrolase<br>**Chain:** B: **PDB Molecule:** sh3b domain-containing protein;<br>**PDBTitle:** sh3-like domain from penicillium virgatum muramidase<br>**PDB Entry:** PDBe RCSB PDBj
74	d2g2xa1	Alignment	not modelled	73.5	22	**Fold:** PUA domain-like<br>**Superfamily:** PUA domain-like<br>**Family:** Atu2648/PH1033-like<br>**PDB entry:** PDBe RCSB PDBj
75	d2evea1	Alignment	not modelled	69.8	22	**Fold:** PUA domain-like<br>**Superfamily:** PUA domain-like<br>**Family:** Atu2648/PH1033-like<br>**PDB entry:** PDBe RCSB PDBj
76	c4pa5A_	Alignment	not modelled	50.5	22	**PDB header:** transferase<br>**Chain:** A: **PDB Molecule:** protein-glutamine gamma-glutamyltransferase;<br>**PDBTitle:** tgl - a bacterial spore coat transglutaminase - cystamine complex<br>**PDB Entry:** PDBe RCSB PDBj
77	c6yexB_	Alignment	not modelled	37.2	32	**PDB header:** hydrolase<br>**Chain:** B: **PDB Molecule:** hnh endonuclease;<br>**PDBTitle:** vcam4i restriction endonuclease in the absence of dna<br>**PDB Entry:** PDBe RCSB PDBj
78	c4eyzB_	Alignment	not modelled	91.4	20	**PDB header:** hydrolase<br>**Chain:** B: **PDB Molecule:** cellulosome-related protein module from ruminococcus<br>**PDBTitle:** crystal structure of an uncommon cellulosome-related protein module2 from ruminococcus flavefaciens that resembles papain-like cysteine3 peptidases<br>**PDB Entry:** PDBe RCSB PDBj
79	c4mhuB_	Alignment	not modelled	35.8	28	**PDB header:** oxidoreductase<br>**Chain:** B: **PDB Molecule:** ectoine hydroxylase;<br>**PDBTitle:** crystal structure of ectd from s. alaskensis with bound fe<br>**PDB Entry:** PDBe RCSB PDBj
80	c7dt0C_	Alignment	not modelled	31.2	28	**PDB header:** hydrolase<br>**Chain:** C: **PDB Molecule:** phytanoyl-coa dioxygenase;<br>**PDBTitle:** proline hydroxylase h11-n101i mutant<br>**PDB Entry:** PDBe RCSB PDBj
81	c2lktA_	Alignment	not modelled	97.1	24	**PDB header:** hydrolase<br>**Chain:** A: **PDB Molecule:** retinoic acid receptor responder protein 3;<br>**PDBTitle:** solution structure of n-terminal domain of human tig3 in 2 m urea<br>**PDB Entry:** PDBe RCSB PDBj
82	c5yboA_	Alignment	not modelled	35.0	24	**PDB header:** oxidoreductase<br>**Chain:** A: **PDB Molecule:** prha;<br>**PDBTitle:** fe(ii)/(alpha)ketoglutarate-dependent dioxygenase prha in complex with2 preaustinoid a1<br>**PDB Entry:** PDBe RCSB PDBj
83	d1ftra1	Alignment	not modelled	26.8	33	**Fold:** Ferredoxin-like<br>**Superfamily:** Formylmethanofuran:tetrahydromethanopterin formyltransferase<br>**Family:** Formylmethanofuran:tetrahydromethanopterin formyltransferase<br>**PDB entry:** PDBe RCSB PDBj
84	d2ba0a2	Alignment	not modelled	21.3	16	**Fold:** Barrel-sandwich hybrid<br>**Superfamily:** Ribosomal L27 protein-like<br>**Family:** ECR1 N-terminal domain-like<br>**PDB entry:** PDBe RCSB PDBj
85	d2if6a1	Alignment	not modelled	97.2	11	**Fold:** Cysteine proteinases<br>**Superfamily:** Cysteine proteinases<br>**Family:** YiiX-like<br>**PDB entry:** PDBe RCSB PDBj
86	c2kytA_	Alignment	not modelled	97.2	18	**PDB header:** hydrolase<br>**Chain:** A: **PDB Molecule:** group xvi phospholipase a2;<br>**PDBTitle:** solution structure of the h-rev107 n-terminal domain<br>**PDB Entry:** PDBe RCSB PDBj
87	c7ddeV_	Alignment	not modelled	42.8	16	**PDB header:** hydrolase<br>**Chain:** V: **PDB Molecule:** aspartyl aminopeptidase 1,zz-type zinc finger-containing<br>**PDBTitle:** cryo-em structure of the ape4 and nbr1 complex<br>**PDB Entry:** PDBe RCSB PDBj
						**PDB header:** hydrolase

88	c3varA_	Alignment	not modelled	37.6	16	**Chain:** A: **PDB Molecule:** aspartyl aminopeptidase;<br>**PDBTitle:** crystal structure of dnpep, znzn form<br>**PDB Entry:** PDBe RCSB PDBj
89	d2nn6h3	Alignment	not modelled	29.0	67	**Fold:** Eukaryotic type KH-domain (KH-domain type I)<br>**Superfamily:** Eukaryotic type KH-domain (KH-domain type I)<br>**Family:** Eukaryotic type KH-domain (KH-domain type I)<br>**PDB entry:** PDBe RCSB PDBj
90	c2opwA_	Alignment	not modelled	50.6	24	**PDB header:** oxidoreductase<br>**Chain:** A: **PDB Molecule:** phyhd1 protein;<br>**PDBTitle:** crystal structure of human phytanoyl-coa dioxygenase phyhd1 (apo)<br>**PDB Entry:** PDBe RCSB PDBj
91	c5zm4B_	Alignment	not modelled	39.4	24	**PDB header:** oxidoreductase<br>**Chain:** B: **PDB Molecule:** dioxygenase anda;<br>**PDBTitle:** fe(ii)/(alpha)ketoglutarate-dependent dioxygenase anda with2 preandiloid c<br>**PDB Entry:** PDBe RCSB PDBj
92	c5epaE_	Alignment	not modelled	34.9	24	**PDB header:** lyase<br>**Chain:** E: **PDB Molecule:** snok;<br>**PDBTitle:** crystal structure of non-heme alpha ketoglutarate dependent2 carbocyclase snok from nogalamycin biosynthesis<br>**PDB Entry:** PDBe RCSB PDBj
93	d2ar1a1	Alignment	not modelled	67.7	26	**Fold:** PUA domain-like<br>**Superfamily:** PUA domain-like<br>**Family:** Atu2648/PH1033-like<br>**PDB entry:** PDBe RCSB PDBj
94	d1igqa_	Alignment	not modelled	46.7	46	**Fold:** SH3-like barrel<br>**Superfamily:** C-terminal domain of transcriptional repressors<br>**Family:** Transcriptional repressor protein KorB<br>**PDB entry:** PDBe RCSB PDBj
95	d1igub_	Alignment	not modelled	46.0	46	**Fold:** SH3-like barrel<br>**Superfamily:** C-terminal domain of transcriptional repressors<br>**Family:** Transcriptional repressor protein KorB<br>**PDB entry:** PDBe RCSB PDBj
96	d2fcta1	Alignment	not modelled	37.8	23	**Fold:** Double-stranded beta-helix<br>**Superfamily:** Clavaminate synthase-like<br>**Family:** PhyH-like<br>**PDB entry:** PDBe RCSB PDBj
97	c6winA_	Alignment	not modelled	21.8	18	**PDB header:** antimicrobial protein<br>**Chain:** A: **PDB Molecule:** type 6 secretion amidase effector 2;<br>**PDBTitle:** type 6 secretion amidase effector 2 (tae2)<br>**PDB Entry:** PDBe RCSB PDBj
98	c3nnlB_	Alignment	not modelled	56.4	16	**PDB header:** biosynthetic protein<br>**Chain:** B: **PDB Molecule:** cura;<br>**PDBTitle:** halogenase domain from cura module (crystal form iii)<br>**PDB Entry:** PDBe RCSB PDBj
99	d1knwa1	Alignment	not modelled	39.8	13	**Fold:** Domain of alpha and beta subunits of F1 ATP synthase-like<br>**Superfamily:** Alanine racemase C-terminal domain-like<br>**Family:** Eukaryotic ODC-like<br>**PDB entry:** PDBe RCSB PDBj
100	c6dchA_	Alignment	not modelled	28.1	28	**PDB header:** oxidoreductase<br>**Chain:** A: **PDB Molecule:** scoe protein;<br>**PDBTitle:** structure of isonitrile biosynthesis enzyme scoe<br>**PDB Entry:** PDBe RCSB PDBj
101	d1twia1	Alignment	not modelled	25.5	21	**Fold:** Domain of alpha and beta subunits of F1 ATP synthase-like<br>**Superfamily:** Alanine racemase C-terminal domain-like<br>**Family:** Eukaryotic ODC-like<br>**PDB entry:** PDBe RCSB PDBj
102	d1m5sa1	Alignment	not modelled	22.3	24	**Fold:** Ferredoxin-like<br>**Superfamily:** Formylmethanofuran:tetrahydromethanopterin formyltransferase<br>**Family:** Formylmethanofuran:tetrahydromethanopterin formyltransferase<br>**PDB entry:** PDBe RCSB PDBj
103	c7vbqB_	Alignment	not modelled	28.9	20	**PDB header:** oxidoreductase<br>**Chain:** B: **PDB Molecule:** fe(ii)/(alpha)ketoglutarate-dependent dioxygenase tlxi;<br>**PDBTitle:** heterodimer structure of fe(ii)/(alpha)ketoglutarate-dependent2 dioxygenase tlxij<br>**PDB Entry:** PDBe RCSB PDBj
104	d2jfga2	Alignment	not modelled	21.3	31	**Fold:** MurD-like peptide ligases, peptide-binding domain<br>**Superfamily:** MurD-like peptide ligases, peptide-binding domain<br>**Family:** MurCDEF C-terminal domain<br>**PDB entry:** PDBe RCSB PDBj
105	c3l6sA_	Alignment	not modelled	38.3	13	**PDB header:** hydrolase<br>**Chain:** A: **PDB Molecule:** aspartyl aminopeptidase;<br>**PDBTitle:** crystal structure of human aspartyl aminopeptidase (dnpep), in complex2 with aspartic acid hydroxamate<br>**PDB Entry:** PDBe RCSB PDBj
106	c4j5iH_	Alignment	not modelled	22.7	38	**PDB header:** oxidoreductase<br>**Chain:** H: **PDB Molecule:** alpha-ketoglutarate-dependent taurine dioxygenase;<br>**PDBTitle:** crystal structure of an alpha-ketoglutarate-dependent taurine2 dioxygenase from mycobacterium smegmatis<br>**PDB Entry:** PDBe RCSB PDBj
107	c2e58D_	Alignment	not modelled	20.6	26	**PDB header:** transferase<br>**Chain:** D: **PDB Molecule:** mnmc2;<br>**PDBTitle:** crystal structure of mnmc2 from aquifex aeolicus<br>**PDB Entry:** PDBe RCSB PDBj
108	c7c9jA_	Alignment	not modelled	58.4	17	**PDB header:** transferase<br>**Chain:** A: **PDB Molecule:** protein-glutamine gamma-glutamyltransferase;<br>**PDBTitle:** transglutaminase from geobacillus stearothermophilus (without c-2 terminal extension)<br>**PDB Entry:** PDBe RCSB PDBj
109	c7qtfA_	Alignment	not modelled	27.1	17	**PDB header:** biosynthetic protein<br>**Chain:** A: **PDB Molecule:** plao1;<br>**PDBTitle:** crystal structure of the fe(ii)/alpha-ketoglutarate dependent2 dioxygenase plao1 in complex with sodium succinate<br>**PDB Entry:** PDBe RCSB PDBj
110	d2zoda2	Alignment	not modelled	26.8	24	**Fold:** PurM C-terminal domain-like<br>**Superfamily:** PurM C-terminal domain-like<br>**Family:** PurM C-terminal domain-like

						**PDB entry:** PDBe RCSB PDBj
111	d1njjb1	Alignment	not modelled	24.0	17	**Fold:** Domain of alpha and beta subunits of F1 ATP synthase-like<br>**Superfamily:** Alanine racemase C-terminal domain-like<br>**Family:** Eukaryotic ODC-like<br>**PDB entry:** PDBe RCSB PDBj
112	d1oiha_	Alignment	not modelled	21.4	31	**Fold:** Double-stranded beta-helix<br>**Superfamily:** Clavaminate synthase-like<br>**Family:** TauD/TfdA-like<br>**PDB entry:** PDBe RCSB PDBj
113	d1wpga1	Alignment	not modelled	25.6	16	**Fold:** Double-stranded beta-helix<br>**Superfamily:** Calcium ATPase, transduction domain A<br>**Family:** Calcium ATPase, transduction domain A<br>**PDB entry:** PDBe RCSB PDBj
114	c3r1jB_	Alignment	not modelled	24.7	33	**PDB header:** oxidoreductase<br>**Chain:** B: **PDB Molecule:** alpha-ketoglutarate-dependent taurine dioxygenase;<br>**PDBTitle:** crystal structure of alpha-ketoglutarate-dependent taurine dioxygenase2 from mycobacterium avium, native form<br>**PDB Entry:** PDBe RCSB PDBj
115	c4ne0A_	Alignment	not modelled	21.4	33	**PDB header:** hydrolase<br>**Chain:** A: **PDB Molecule:** l-arginine beta-hydroxylase;<br>**PDBTitle:** crystal structure of non-heme iron oxygenase orfp d157a mutant in2 complex with (3s)-hydroxy-l-arg<br>**PDB Entry:** PDBe RCSB PDBj
116	c4ne0B_	Alignment	not modelled	20.7	36	**PDB header:** hydrolase<br>**Chain:** B: **PDB Molecule:** l-arginine beta-hydroxylase;<br>**PDBTitle:** crystal structure of non-heme iron oxygenase orfp d157a mutant in2 complex with (3s)-hydroxy-l-arg<br>**PDB Entry:** PDBe RCSB PDBj
117	c7tclX_	Alignment	not modelled	20.5	33	**PDB header:** oxidoreductase<br>**Chain:** X: **PDB Molecule:** taud/tfda family dioxygenase;<br>**PDBTitle:** crystal structure of p.isnb complexed with tyrosine isonitrile<br>**PDB Entry:** PDBe RCSB PDBj
118	c2vpmB_	Alignment	not modelled	87.6	11	**PDB header:** ligase<br>**Chain:** B: **PDB Molecule:** trypanothione synthetase;<br>**PDBTitle:** trypanothione synthetase<br>**PDB Entry:** PDBe RCSB PDBj
119	c5vn6B_	Alignment	not modelled	22.8	25	**PDB header:** oxidoreductase<br>**Chain:** B: **PDB Molecule:** taurine dioxygenase;<br>**PDBTitle:** crystal structure of taurine dioxygenase from burkholderia ambifaria<br>**PDB Entry:** PDBe RCSB PDBj
120	d1ds1a_	Alignment	not modelled	22.1	25	**Fold:** Double-stranded beta-helix<br>**Superfamily:** Clavaminate synthase-like<br>**Family:** Clavaminate synthase<br>**PDB entry:** PDBe RCSB PDBj
