# Supplementary material for: Characterization of Two Novel Endolysins from Bacteriophage PEF1 and Evaluation of Their Combined Effects on the Control of Enterococcus faecalis Planktonic and Biofilm Cells
Source: Antibiotics (Basel). 2024 Sep 13;13(9):884. doi: 10.3390/antibiotics13090884 (PMC11428236; doi:10.3390/antibiotics13090884)
Supplement: Supplementary file 1 [file antibiotics-13-00884-s001.zip › Table S3.pdf]

Table S3: Three-dimensional alignment results of LysPEF1-2 with similar protein templates.

Phyre2

Email	wangchen950518@gmail.com
Description	LysPEF1-2
Date	Wed Aug 21 05:45:36 BST 2024
Unique Job ID	f26785b1c5a04027

Detailed template information

#	Template	Alignment Coverage	3D Model	Confidence	% i.d.	Template Information
1	c4bxdB_	Alignment	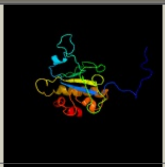	100.0	28	**PDB header:** hydrolase/peptide **Chain:** B: **PDB Molecule:** ampdh3; **PDBTitle:** crystal structure of ampdh3 from pseudomonas aeruginosa in complex2 with tetrasaccharide pentapeptide **PDB Entry:** PDBe RCSB PDBj
2	c4bolA_	Alignment	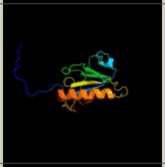	100.0	30	**PDB header:** hydrolase **Chain:** A: **PDB Molecule:** ampdh2; **PDBTitle:** crystal structure of ampdh2 from pseudomonas aeruginosa in complex2 with pentapeptide **PDB Entry:** PDBe RCSB PDBj
3	c4olsA_	Alignment	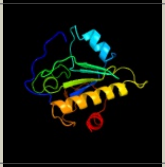	100.0	27	**PDB header:** hydrolase **Chain:** A: **PDB Molecule:** endolysin; **PDBTitle:** the amidase-2 domain of lysgh15 **PDB Entry:** PDBe RCSB PDBj
4	c8c4dA_	Alignment	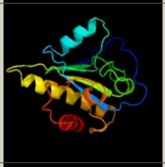	100.0	23	**PDB header:** antimicrobial protein **Chain:** A: **PDB Molecule:** n-acetylmuramoyl-l-alanine amidase; **PDBTitle:** n-acetylmuramoyl-l-alanine amidase from enterococcus faecium prophage2 genome **PDB Entry:** PDBe RCSB PDBj
5	c3hmaA_	Alignment	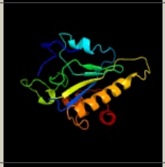	100.0	24	**PDB header:** hydrolase **Chain:** A: **PDB Molecule:** n-acetylmuramoyl-l-alanine amidase xlya; **PDBTitle:** amidase from bacillus subtilis **PDB Entry:** PDBe RCSB PDBj
6	c2bh7A_	Alignment	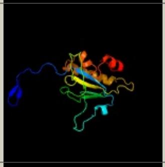	100.0	23	**PDB header:** hydrolase **Chain:** A: **PDB Molecule:** n-acetylmuramoyl-l-alanine amidase; **PDBTitle:** crystal structure of a semet derivative of amid at 2.22 angstroms **PDB Entry:** PDBe RCSB PDBj
7	d2bgxa2	Alignment	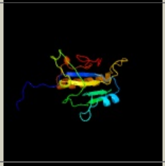	100.0	26	**Fold:** N-acetylmuramoyl-L-alanine amidase-like **Superfamily:** N-acetylmuramoyl-L-alanine amidase-like **Family:** N-acetylmuramoyl-L-alanine amidase-like **PDB entry:** PDBe RCSB PDBj
8	c6sscA_	Alignment	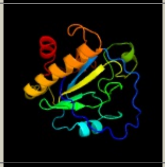	100.0	22	**PDB header:** hydrolase **Chain:** A: **PDB Molecule:** n-acetylmuramoyl-l-alanine amidase; **PDBTitle:** n-acetylmuramoyl-l-alanine amidase lysc from clostridium intestinale2 urnw **PDB Entry:** PDBe RCSB PDBj
9	c3latB_	Alignment	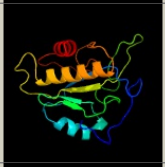	100.0	20	**PDB header:** hydrolase **Chain:** B: **PDB Molecule:** bifunctional autolysin; **PDBTitle:** crystal structure of staphylococcus peptidoglycan hydrolase amie **PDB Entry:** PDBe RCSB PDBj
10	c1ohtA_	Alignment	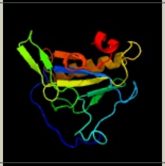	99.9	20	**PDB header:** hydrolase **Chain:** A: **PDB Molecule:** cg14704 protein; **PDBTitle:** peptidoglycan recognition protein-lb **PDB Entry:** PDBe RCSB PDBj
11	d1ohta_	Alignment	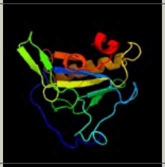	99.9	20	**Fold:** N-acetylmuramoyl-L-alanine amidase-like **Superfamily:** N-acetylmuramoyl-L-alanine amidase-like **Family:** N-acetylmuramoyl-L-alanine amidase-like **PDB entry:** PDBe RCSB PDBj

12	c4yu5A_	Alignment	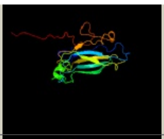	62.6	18	**PDB header:** hydrolase<br>**Chain:** A: **PDB Molecule:** immune inhibitor a, metalloprotease;<br>**PDBTitle:** crystal structure of selenomethionine variant of bacillus anthracis2 immune inhibitor a2 peptidase zymogen<br>**PDB Entry:** PDBe RCSB PDBj
13	d1lbaa_	Alignment	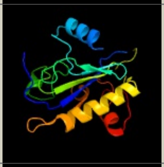	100.0	19	**Fold:** N-acetylmuramoyl-L-alanine amidase-like<br>**Superfamily:** N-acetylmuramoyl-L-alanine amidase-like<br>**Family:** N-acetylmuramoyl-L-alanine amidase-like<br>**PDB entry:** PDBe RCSB PDBj
14	d1j3ga_	Alignment	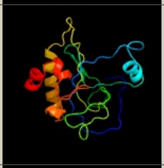	100.0	18	**Fold:** N-acetylmuramoyl-L-alanine amidase-like<br>**Superfamily:** N-acetylmuramoyl-L-alanine amidase-like<br>**Family:** N-acetylmuramoyl-L-alanine amidase-like<br>**PDB entry:** PDBe RCSB PDBj
15	d1yb0a1	Alignment	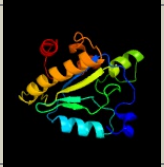	100.0	18	**Fold:** N-acetylmuramoyl-L-alanine amidase-like<br>**Superfamily:** N-acetylmuramoyl-L-alanine amidase-like<br>**Family:** N-acetylmuramoyl-L-alanine amidase-like<br>**PDB entry:** PDBe RCSB PDBj
16	c6fhgB_	Alignment	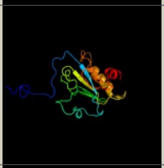	99.9	18	**PDB header:** antimicrobial protein<br>**Chain:** B: **PDB Molecule:** lyst endolysin;<br>**PDBTitle:** crystal structure of the ts2631 endolysin from thermus scotoductus2 phage with the unique n-terminal moiety responsible for peptidoglycan3 anchoring<br>**PDB Entry:** PDBe RCSB PDBj
17	d2cb3a1	Alignment	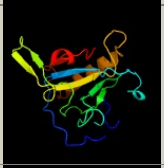	100.0	18	**Fold:** N-acetylmuramoyl-L-alanine amidase-like<br>**Superfamily:** N-acetylmuramoyl-L-alanine amidase-like<br>**Family:** N-acetylmuramoyl-L-alanine amidase-like<br>**PDB entry:** PDBe RCSB PDBj
18	c4z8iA_	Alignment	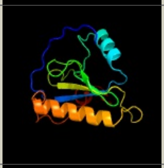	99.9	19	**PDB header:** hydrolase<br>**Chain:** A: **PDB Molecule:** peptidoglycan recognition protein 3;<br>**PDBTitle:** crystal structure of branchiostoma belcheri tsingtauense peptidoglycan2 recognition protein 3<br>**PDB Entry:** PDBe RCSB PDBj
19	c2rkqA_	Alignment	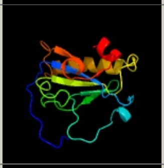	100.0	18	**PDB header:** immune system<br>**Chain:** A: **PDB Molecule:** peptidoglycan-recognition protein-sd;<br>**PDBTitle:** crystal structure of drosophila peptidoglycan recognition protein sd2 (pgrp-sd)<br>**PDB Entry:** PDBe RCSB PDBj
20	c7f5iA_	Alignment	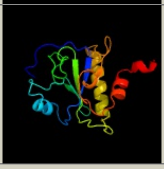	100.0	17	**PDB header:** hydrolase<br>**Chain:** A: **PDB Molecule:** amidase;<br>**PDBTitle:** x-ray structure of clostridium perfringens-specific amidase endolysin<br>**PDB Entry:** PDBe RCSB PDBj
21	d1ycka1	Alignment	not modelled	100.0	17	**Fold:** N-acetylmuramoyl-L-alanine amidase-like<br>**Superfamily:** N-acetylmuramoyl-L-alanine amidase-like<br>**Family:** N-acetylmuramoyl-L-alanine amidase-like<br>**PDB entry:** PDBe RCSB PDBj
22	c5xz3D_	Alignment	not modelled	99.9	17	**PDB header:** immune system<br>**Chain:** D: **PDB Molecule:** peptidoglycan-recognition protein;<br>**PDBTitle:** the x-ray structure of apis mellifera pgrp-sa<br>**PDB Entry:** PDBe RCSB PDBj
23	d2f2lx1	Alignment	not modelled	99.9	18	**Fold:** N-acetylmuramoyl-L-alanine amidase-like<br>**Superfamily:** N-acetylmuramoyl-L-alanine amidase-like<br>**Family:** N-acetylmuramoyl-L-alanine amidase-like<br>**PDB entry:** PDBe RCSB PDBj
24	d1sk4a_	Alignment	not modelled	99.9	17	**Fold:** N-acetylmuramoyl-L-alanine amidase-like<br>**Superfamily:** N-acetylmuramoyl-L-alanine amidase-like<br>**Family:** N-acetylmuramoyl-L-alanine amidase-like<br>**PDB entry:** PDBe RCSB PDBj
25	c2xz4A_	Alignment	not modelled	100.0	17	**PDB header:** immune system<br>**Chain:** A: **PDB Molecule:** peptidoglycan-recognition protein lf;<br>**PDBTitle:** crystal structure of the lfz ectodomain of the peptidoglycan2 recognition protein lf<br>**PDB Entry:** PDBe RCSB PDBj
26	c3ep1B_	Alignment	not modelled	99.9	15	**PDB header:** immune system<br>**Chain:** B: **PDB Molecule:** pgrp-hd - peptidoglycan recognition protein homologue;<br>**PDBTitle:** structure of the pgrp-hd from alvinella pompejana<br>**PDB Entry:** PDBe RCSB PDBj
27	d1sxra_	Alignment	not modelled	99.9	13	**Fold:** N-acetylmuramoyl-L-alanine amidase-like<br>**Superfamily:** N-acetylmuramoyl-L-alanine amidase-like<br>**Family:** N-acetylmuramoyl-L-alanine amidase-like<br>**PDB entry:** PDBe RCSB PDBj
28	c6ckhA_	Alignment	not modelled	99.9	14	**PDB header:** immune system<br>**Chain:** A: **PDB Molecule:** peptidoglycan-recognition protein;<br>**PDBTitle:** manduca sexta peptidoglycan recognition protein-1<br>**PDB Entry:** PDBe RCSB PDBj

29	c1s2jA_	Alignment	not modelled	99.9	14	**PDB header:** hydrolase<br>**Chain:** A: **PDB Molecule:** peptidoglycan recognition protein sa cg11709-pa;<br>**PDBTitle:** crystal structure of the drosophila pattern-recognition receptor pgrp-2 sa<br>**PDB Entry:** PDBe RCSB PDBj
30	c2os2A_	Alignment	not modelled	26.0	31	**PDB header:** oxidoreductase<br>**Chain:** A: **PDB Molecule:** jmjc domain-containing histone demethylation protein 3a;<br>**PDBTitle:** crystal structure of jmjd2a complexed with histone h3 peptide2 trimethylated at lys36<br>**PDB Entry:** PDBe RCSB PDBj
31	c4ivvA_	Alignment	not modelled	100.0	11	**PDB header:** hydrolase<br>**Chain:** A: **PDB Molecule:** autolysin;<br>**PDBTitle:** catalytic amidase domain of the major autolysin lyta from2 streptococcus pneumaniae<br>**PDB Entry:** PDBe RCSB PDBj
32	c4x36A_	Alignment	not modelled	99.8	11	**PDB header:** hydrolase<br>**Chain:** A: **PDB Molecule:** autolysin;<br>**PDBTitle:** crystal structure of the autolysin lyta from streptococcus pneumoniae2 tigr4<br>**PDB Entry:** PDBe RCSB PDBj
33	c5iklD_	Alignment	not modelled	18.3	21	**PDB header:** ligase<br>**Chain:** D: **PDB Molecule:** geranyl-coa carboxylase, beta-subunit;<br>**PDBTitle:** crystal structure of p. aeruginosa geranyl-coa carboxylase (gcc), beta2 subunit<br>**PDB Entry:** PDBe RCSB PDBj
34	c7eebJ_	Alignment	not modelled	47.9	14	**PDB header:** protein transport<br>**Chain:** J: **PDB Molecule:** transmembrane protein 249;<br>**PDBTitle:** structure of the catspermasome<br>**PDB Entry:** PDBe RCSB PDBj
35	c6cuqB_	Alignment	not modelled	15.8	21	**PDB header:** cytokine<br>**Chain:** B: **PDB Molecule:** macrophage migration inhibitory factor-like protein;<br>**PDBTitle:** crystal structure of macrophage migration inhibitory factor-like2 protein (ehmif) from entamoeba histolytica<br>**PDB Entry:** PDBe RCSB PDBj
36	c4atbB_	Alignment	not modelled	14.4	22	**PDB header:** immune system<br>**Chain:** B: **PDB Molecule:** interleukin enhancer-binding factor 3;<br>**PDBTitle:** crystal structure of the nf90-nf45 dimerisation domain complex with2 ctp<br>**PDB Entry:** PDBe RCSB PDBj
37	d1vk3a2	Alignment	not modelled	28.7	19	**Fold:** Bacillus chorismate mutase-like<br>**Superfamily:** PurM N-terminal domain-like<br>**Family:** PurM N-terminal domain-like<br>**PDB entry:** PDBe RCSB PDBj
38	d2dsta1	Alignment	not modelled	16.7	14	**Fold:** alpha/beta-Hydrolases<br>**Superfamily:** alpha/beta-Hydrolases<br>**Family:** TTHA1544-like<br>**PDB entry:** PDBe RCSB PDBj
39	c6u2mC_	Alignment	not modelled	28.2	12	**PDB header:** hydrolase<br>**Chain:** C: **PDB Molecule:** halocamp v2;<br>**PDBTitle:** crystal structure of a halotag-based calcium indicator, halocamp v2,2 bound to jf635<br>**PDB Entry:** PDBe RCSB PDBj
40	d2f2la1	Alignment	not modelled	99.9	8	**Fold:** N-acetylmuramoyl-L-alanine amidase-like<br>**Superfamily:** N-acetylmuramoyl-L-alanine amidase-like<br>**Family:** N-acetylmuramoyl-L-alanine amidase-like<br>**PDB entry:** PDBe RCSB PDBj
41	d1sxea_	Alignment	not modelled	13.9	21	**Fold:** SAM domain-like<br>**Superfamily:** SAM/Pointed domain<br>**Family:** Pointed domain<br>**PDB entry:** PDBe RCSB PDBj
42	c2xz8A_	Alignment	not modelled	99.1	10	**PDB header:** immune system<br>**Chain:** A: **PDB Molecule:** peptidoglycan-recognition protein lf;<br>**PDBTitle:** crystal structure of the lfw ectodomain of the peptidoglycan2 recognition protein lf<br>**PDB Entry:** PDBe RCSB PDBj
43	c2os5C_	Alignment	not modelled	22.3	17	**PDB header:** cytokine<br>**Chain:** C: **PDB Molecule:** acemif;<br>**PDBTitle:** macrophage migration inhibitory factor from ancylostoma ceylanicum<br>**PDB Entry:** PDBe RCSB PDBj
44	d1e0ga_	Alignment	not modelled	19.0	34	**Fold:** LysM domain<br>**Superfamily:** LysM domain<br>**Family:** LysM domain<br>**PDB entry:** PDBe RCSB PDBj
45	d1dpta_	Alignment	not modelled	24.5	14	**Fold:** Tautomerase/MIF<br>**Superfamily:** Tautomerase/MIF<br>**Family:** MIF-related<br>**PDB entry:** PDBe RCSB PDBj
46	c3b64A_	Alignment	not modelled	47.9	22	**PDB header:** cytokine<br>**Chain:** A: **PDB Molecule:** macrophage migration inhibitory factor-like protein;<br>**PDBTitle:** macrophage migration inhibitory factor (mif) from /leishmania major<br>**PDB Entry:** PDBe RCSB PDBj
47	d1hfoa_	Alignment	not modelled	33.6	22	**Fold:** Tautomerase/MIF<br>**Superfamily:** Tautomerase/MIF<br>**Family:** MIF-related<br>**PDB entry:** PDBe RCSB PDBj
48	c8ap3A_	Alignment	not modelled	24.5	22	**PDB header:** cytokine<br>**Chain:** A: **PDB Molecule:** mif homologue;<br>**PDBTitle:** structure of a. thaliana mif/d-dt-like protein-2 (mdl2)<br>**PDB Entry:** PDBe RCSB PDBj
49	c7cigB_	Alignment	not modelled	25.5	28	**PDB header:** lyase<br>**Chain:** B: **PDB Molecule:** l-methionine decarboxylase;<br>**PDBTitle:** crystal structure of l-methionine decarboxylase q64a mutant from2 streptomyces sp.590 in complexed with l-methionine methyl ester3 (geminal diamine form).<br>**PDB Entry:** PDBe RCSB PDBj
						**PDB header:** cytokine<br>**Chain:** A: **PDB Molecule:** macrophage migration inhibitory factor-

50	c3fwtA_	Alignment	not modelled	51.9	30	like protein;<br>**PDBTitle:** crystal structure of leishmania major mif2<br>**PDB Entry:** PDBe RCSB PDBj
51	c2wpvG_	Alignment	not modelled	32.5	15	**PDB header:** protein binding<br>**Chain:** G: **PDB Molecule:** upf0363 protein yor164c;<br>**PDBTitle:** crystal structure of s. cerevisiae get4-get5 complex<br>**PDB Entry:** PDBe RCSB PDBj
52	c2gwoC_	Alignment	not modelled	14.5	14	**PDB header:** hydrolase<br>**Chain:** C: **PDB Molecule:** dual specificity protein phosphatase 13;<br>**PDBTitle:** crystal structure of tmdp<br>**PDB Entry:** PDBe RCSB PDBj
53	d3b5ea1	Alignment	not modelled	20.0	23	**Fold:** alpha/beta-Hydrolases<br>**Superfamily:** alpha/beta-Hydrolases<br>**Family:** Carboxylesterase/thioesterase 1<br>**PDB entry:** PDBe RCSB PDBj
54	d1lpbb2	Alignment	not modelled	50.9	26	**Fold:** alpha/beta-Hydrolases<br>**Superfamily:** alpha/beta-Hydrolases<br>**Family:** Pancreatic lipase, N-terminal domain<br>**PDB entry:** PDBe RCSB PDBj
55	d1bu8a2	Alignment	not modelled	50.8	26	**Fold:** alpha/beta-Hydrolases<br>**Superfamily:** alpha/beta-Hydrolases<br>**Family:** Pancreatic lipase, N-terminal domain<br>**PDB entry:** PDBe RCSB PDBj
56	d1etha2	Alignment	not modelled	49.1	26	**Fold:** alpha/beta-Hydrolases<br>**Superfamily:** alpha/beta-Hydrolases<br>**Family:** Pancreatic lipase, N-terminal domain<br>**PDB entry:** PDBe RCSB PDBj
57	c6e7kB_	Alignment	not modelled	47.9	26	**PDB header:** hydrolase<br>**Chain:** B: **PDB Molecule:** lipoprotein lipase;<br>**PDBTitle:** structure of the lipoprotein lipase gpihbp1 complex that mediates2 plasma triglyceride hydrolysis<br>**PDB Entry:** PDBe RCSB PDBj
58	c2pvsB_	Alignment	not modelled	47.5	26	**PDB header:** hydrolase<br>**Chain:** B: **PDB Molecule:** pancreatic lipase-related protein 2;<br>**PDBTitle:** structure of human pancreatic lipase related protein 2 mutant n336q<br>**PDB Entry:** PDBe RCSB PDBj
59	c3d0kA_	Alignment	not modelled	17.6	25	**PDB header:** hydrolase<br>**Chain:** A: **PDB Molecule:** putative poly(3-hydroxybutyrate) depolymerase lpqc;<br>**PDBTitle:** crystal structure of the lpqc, poly(3-hydroxybutyrate) depolymerase2 from bordetella parapertussis<br>**PDB Entry:** PDBe RCSB PDBj
60	c8dtlD_	Alignment	not modelled	13.5	35	**PDB header:** signaling protein<br>**Chain:** D: **PDB Molecule:** insulin mimetic peptide s597;<br>**PDBTitle:** cryo-em structure of insulin receptor (ir) bound with s597 peptide<br>**PDB Entry:** PDBe RCSB PDBj
61	c8dtlC_	Alignment	not modelled	13.5	35	**PDB header:** signaling protein<br>**Chain:** C: **PDB Molecule:** insulin mimetic peptide s597;<br>**PDBTitle:** cryo-em structure of insulin receptor (ir) bound with s597 peptide<br>**PDB Entry:** PDBe RCSB PDBj
62	c8h92A_	Alignment	not modelled	14.1	14	**PDB header:** plant protein<br>**Chain:** A: **PDB Molecule:** triphosphate tunel metalloenzyme 3-like;<br>**PDBTitle:** ziziphus jujuba adenylyl cyclase<br>**PDB Entry:** PDBe RCSB PDBj
63	c1djyB_	Alignment	not modelled	15.7	13	**PDB header:** lipid degradation<br>**Chain:** B: **PDB Molecule:** phosphoinositide-specific phospholipase c, isozyme delta1;<br>**PDBTitle:** phosphoinositide-specific phospholipase c-delta1 from rat complexed2 with inositol-2,4,5-trisphosphate<br>**PDB Entry:** PDBe RCSB PDBj
64	c6i28A_	Alignment	not modelled	29.0	14	**PDB header:** viral protein<br>**Chain:** A: **PDB Molecule:** orf98 ptp-2;<br>**PDBTitle:** crystal structure of cydia pomonella ptp-2 phosphatase<br>**PDB Entry:** PDBe RCSB PDBj
65	c6lkvF_	Alignment	not modelled	72.8	10	**PDB header:** cytokine<br>**Chain:** F: **PDB Molecule:** macrophage migration inhibitory factor;<br>**PDBTitle:** structural and functional insights into macrophage migration2 inhibitory factor from oncomelania hupensis, the intermediate host of3 schistosoma japonicum<br>**PDB Entry:** PDBe RCSB PDBj
66	c7c7lB_	Alignment	not modelled	19.2	19	**PDB header:** rna binding protein/rna/dna<br>**Chain:** B: **PDB Molecule:** crispr-associated protein cas14a.1;<br>**PDBTitle:** cryo-em structure of the cas12f1-sgrna-target dna complex<br>**PDB Entry:** PDBe RCSB PDBj
67	c4p7nA_	Alignment	not modelled	13.8	19	**PDB header:** hydrolase<br>**Chain:** A: **PDB Molecule:** poly-beta-1,6-n-acetyl-d-glucosamine n-deacetylase;<br>**PDBTitle:** structure of escherichia coli pgab c-terminal domain in complex with2 glucosamine<br>**PDB Entry:** PDBe RCSB PDBj
68	c6culG_	Alignment	not modelled	36.2	25	**PDB header:** transferase<br>**Chain:** G: **PDB Molecule:** pyoverdine synthetase f;<br>**PDBTitle:** pvdf of pyoverdin biosynthesis is a structurally unique n10-2 formyltetrahydrofolate-dependent formyltransferase<br>**PDB Entry:** PDBe RCSB PDBj
69	c4as2D_	Alignment	not modelled	13.7	30	**PDB header:** hydrolase<br>**Chain:** D: **PDB Molecule:** phosphorylcholine phosphatase;<br>**PDBTitle:** pseudomonas aeruginosa phosphorylcholine phosphatase. monoclinic form<br>**PDB Entry:** PDBe RCSB PDBj
70	d1gd0a_	Alignment	not modelled	45.7	23	**Fold:** Tautomerase/MIF<br>**Superfamily:** Tautomerase/MIF<br>**Family:** MIF-related<br>**PDB entry:** PDBe RCSB PDBj
71	d2gdga1	Alignment	not modelled	41.7	23	**Fold:** Tautomerase/MIF<br>**Superfamily:** Tautomerase/MIF<br>**Family:** MIF-related<br>**PDB entry:** PDBe RCSB PDBj

72	d1fima_	Alignment	not modelled	38.0	23	**Fold:** Tautomerase/MIF<br>**Superfamily:** Tautomerase/MIF<br>**Family:** MIF-related<br>**PDB entry:** PDBe RCSB PDBj
73	c8sl7A_	Alignment	not modelled	68.4	18	**PDB header:** lyase<br>**Chain:** A: **PDB Molecule:** tryptophanase;<br>**PDBTitle:** butyricicoccus sp. bioml-a1 tryptophanase complex with (3s) alg-05<br>**PDB Entry:** PDBe RCSB PDBj
74	c2pplA_	Alignment	not modelled	52.0	22	**PDB header:** hydrolase<br>**Chain:** A: **PDB Molecule:** pancreatic lipase-related protein 1;<br>**PDBTitle:** human pancreatic lipase-related protein 1<br>**PDB Entry:** PDBe RCSB PDBj
75	c1hplB_	Alignment	not modelled	51.8	22	**PDB header:** hydrolase(carboxylic esterase)<br>**Chain:** B: **PDB Molecule:** lipase;<br>**PDBTitle:** horse pancreatic lipase. the crystal structure at 2.3 angstroms2 resolution<br>**PDB Entry:** PDBe RCSB PDBj
76	d1hpla2	Alignment	not modelled	50.2	22	**Fold:** alpha/beta-Hydrolases<br>**Superfamily:** alpha/beta-Hydrolases<br>**Family:** Pancreatic lipase, N-terminal domain<br>**PDB entry:** PDBe RCSB PDBj
77	c1gplA_	Alignment	not modelled	49.5	22	**PDB header:** serine esterase<br>**Chain:** A: **PDB Molecule:** rp2 lipase;<br>**PDBTitle:** rp2 lipase<br>**PDB Entry:** PDBe RCSB PDBj
78	d1gpla2	Alignment	not modelled	39.1	19	**Fold:** alpha/beta-Hydrolases<br>**Superfamily:** alpha/beta-Hydrolases<br>**Family:** Pancreatic lipase, N-terminal domain<br>**PDB entry:** PDBe RCSB PDBj
79	d2hfda1	Alignment	not modelled	14.8	10	**Fold:** Thioredoxin fold<br>**Superfamily:** Thioredoxin-like<br>**Family:** HyaE-like<br>**PDB entry:** PDBe RCSB PDBj
80	c3gacD_	Alignment	not modelled	17.8	14	**PDB header:** cytokine<br>**Chain:** D: **PDB Molecule:** macrophage migration inhibitory factor-like protein;<br>**PDBTitle:** structure of mif with hpp<br>**PDB Entry:** PDBe RCSB PDBj
81	c4qnnC_	Alignment	not modelled	47.9	19	**PDB header:** hydrolase<br>**Chain:** C: **PDB Molecule:** phospholipase a 1 from hornet(vespa basalis) venom;<br>**PDBTitle:** crystal structure of phospholipase a 1 from hornet(vespa basalis)2 venom<br>**PDB Entry:** PDBe RCSB PDBj
82	c3b79A_	Alignment	not modelled	38.4	11	**PDB header:** nucleotide binding protein<br>**Chain:** A: **PDB Molecule:** toxin secretion atp-binding protein;<br>**PDBTitle:** crystal structure of the n-terminal peptidase c39 like2 domain of the toxin secretion atp-binding protein from3 vibrio parahaemolyticus<br>**PDB Entry:** PDBe RCSB PDBj
83	c6jppA_	Alignment	not modelled	35.0	11	**PDB header:** signaling protein<br>**Chain:** A: **PDB Molecule:** engulfment and cell motility protein 1;<br>**PDBTitle:** solution structure of elmo1 rbd<br>**PDB Entry:** PDBe RCSB PDBj
84	c2p4dA_	Alignment	not modelled	13.6	10	**PDB header:** hydrolase<br>**Chain:** A: **PDB Molecule:** dual specificity protein phosphatase;<br>**PDBTitle:** structure-assisted discovery of variola major h1 phosphatase2 inhibitors<br>**PDB Entry:** PDBe RCSB PDBj
85	c5xyiM_	Alignment	not modelled	13.9	29	**PDB header:** ribosome<br>**Chain:** M: **PDB Molecule:** ribosomal protein l7ae, putative;<br>**PDBTitle:** small subunit of trichomonas vaginalis ribosome<br>**PDB Entry:** PDBe RCSB PDBj
86	c5xjvA_	Alignment	not modelled	13.9	11	**PDB header:** hydrolase<br>**Chain:** A: **PDB Molecule:** dual specificity protein phosphatase 13 isoform a;<br>**PDBTitle:** two intermediate states of conformation switch in dual specificity2 phosphatase 13a<br>**PDB Entry:** PDBe RCSB PDBj
87	c8dq6A_	Alignment	not modelled	38.6	11	**PDB header:** cytokine<br>**Chain:** A: **PDB Molecule:** mif/d-dt-like protein-1;<br>**PDBTitle:** structure of a. thaliana mif/d-dt-like protein-1 (mdl1)<br>**PDB Entry:** PDBe RCSB PDBj
88	c1rp1A_	Alignment	not modelled	48.0	15	**PDB header:** hydrolase<br>**Chain:** A: **PDB Molecule:** pancreatic lipase related protein 1;<br>**PDBTitle:** dog pancreatic lipase related protein 1<br>**PDB Entry:** PDBe RCSB PDBj
89	d1rp1a2	Alignment	not modelled	47.9	15	**Fold:** alpha/beta-Hydrolases<br>**Superfamily:** alpha/beta-Hydrolases<br>**Family:** Pancreatic lipase, N-terminal domain<br>**PDB entry:** PDBe RCSB PDBj
90	c2rpsA_	Alignment	not modelled	15.6	57	**PDB header:** immune system<br>**Chain:** A: **PDB Molecule:** chemokine;<br>**PDBTitle:** solution structure of a novel insect chemokine isolated from2 integument<br>**PDB Entry:** PDBe RCSB PDBj
91	c8vj2C_	Alignment	not modelled	48.4	18	**PDB header:** cytokine<br>**Chain:** C: **PDB Molecule:** macrophage migration inhibitory factor-1;<br>**PDBTitle:** crystal structure of macrophage migration inhibitory factor-1 (mif1)2 from onchocerca volvulus<br>**PDB Entry:** PDBe RCSB PDBj
92	c2zycA_	Alignment	not modelled	14.1	18	**PDB header:** hydrolase<br>**Chain:** A: **PDB Molecule:** peptidoglycan hydrolase flgj;<br>**PDBTitle:** crystal structure of peptidoglycan hydrolase from sphingomonas sp. a1<br>**PDB Entry:** PDBe RCSB PDBj
93	c2y96A_	Alignment	not modelled	13.6	9	**PDB header:** hydrolase<br>**Chain:** A: **PDB Molecule:** dual specificity phosphatase dupd1;<br>**PDBTitle:** structure of human dual-specificity phosphatase 27<br>**PDB Entry:** PDBe RCSB PDBj
						**PDB header:** immune system

94	c3t5sA_	Alignment	not modelled	54.6	14	**Chain:** A: **PDB Molecule:** macrophage migration inhibitory factor;<br>**PDBTitle:** structure of macrophage migration inhibitory factor from giardia2 lamblia<br>**PDB Entry:** PDBe RCSB PDBj
95	c8ur2E_	Alignment	not modelled	45.0	11	**PDB header:** cytokine<br>**Chain:** E: **PDB Molecule:** macrophage migration inhibitory factor;<br>**PDBTitle:** crystal structure of macrophage migration inhibitory factor (mif) from2 trichomonas vaginalis (i41 form)<br>**PDB Entry:** PDBe RCSB PDBj
96	c8dqaA_	Alignment	not modelled	26.8	10	**PDB header:** cytokine<br>**Chain:** A: **PDB Molecule:** ls1-like protein;<br>**PDBTitle:** structure of a. thaliana mif/d-dt-like protein-3 (mdl3)<br>**PDB Entry:** PDBe RCSB PDBj
97	c3zuaA_	Alignment	not modelled	14.3	6	**PDB header:** hydrolase<br>**Chain:** A: **PDB Molecule:** alpha-hemolysin translocation atp-binding protein hlyb;<br>**PDBTitle:** a c39-like domain<br>**PDB Entry:** PDBe RCSB PDBj
98	c3og9A_	Alignment	not modelled	14.3	6	**PDB header:** hydrolase<br>**Chain:** A: **PDB Molecule:** protein yahd a copper inducible hydrolase;<br>**PDBTitle:** structure of yahd with malic acid<br>**PDB Entry:** PDBe RCSB PDBj
99	c2q0xA_	Alignment	not modelled	27.7	4	**PDB header:** structural genomics, unknown function<br>**Chain:** A: **PDB Molecule:** uncharacterized protein;<br>**PDBTitle:** alpha/beta hydrolase fold protein of unknown function<br>**PDB Entry:** PDBe RCSB PDBj
